# Author Correction: Spatial communication systems across languages reflect universal action constraints

**DOI:** 10.1038/s41562-023-01806-3

**Published:** 2024-01-09

**Authors:** Kenny R. Coventry, Harmen B. Gudde, Holger Diessel, Jacqueline Collier, Pedro Guijarro-Fuentes, Mila Vulchanova, Valentin Vulchanov, Emanuela Todisco, Maria Reile, Merlijn Breunesse, Helen Plado, Juergen Bohnemeyer, Raed Bsili, Michela Caldano, Rositsa Dekova, Katharine Donelson, Diana Forker, Yesol Park, Lekhnath Sharma Pathak, David Peeters, Gabriella Pizzuto, Baris Serhan, Linda Apse, Florian Hesse, Linh Hoang, Phuong Hoang, Yoko Igari, Keerthana Kapiley, Tamar Haupt-Khutsishvili, Sara Kolding, Katri Priiki, Ieva Mačiukaitytė, Vaisnavi Mohite, Tiina Nahkola, Sum Yi Tsoi, Stefan Williams, Shunei Yasuda, Angelo Cangelosi, Jon Andoni Duñabeitia, Ramesh Kumar Mishra, Roberta Rocca, Jurģis Šķilters, Mikkel Wallentin, Eglė Žilinskaitė-Šinkūnienė, Ozlem Durmaz Incel

**Affiliations:** 1 School of Psychology, University of East Anglia, Norwich, UK; 2 Helmholtz Institute, Department of Experimental Psychology, Utrecht University, Utrecht, the Netherlands; 3 Hanse-Wissenschaftskolleg, Delmenhorst, Germany; 4 Department of English, Friedrich-Schiller-Universität Jena, Jena, Germany; 5 Department of Spanish, Modern and Classic Philology, University of the Balearic Islands, Palma, Spain; 6 Language Acquisition and Language Processing Lab, Department of Language and Literature, Norwegian University of Science and Technology, Trondheim, Norway; 7 Department of Spanish Language, Linguistics and Literature Theory, University of Seville, Seville, Spain; 8 Institute of Estonian and General Linguistics, University of Tartu, Tartu, Estonia; 9 Centre for the Arts in Society, Leiden University, Leiden, the Netherlands; 10 Võro Institute, Võru, Estonia; 11 Department of Linguistics, University at Buffalo, Buffalo, NY USA; 12 Danieli Telerobot Srl, Genoa, Italy; 13 Istituto Italiano di Tecnologia (IIT), Genoa, Italy; 14 Paisii Hilendarski University of Plovdiv, Plovdiv, Bulgaria; 15 Department of English, University of Nevada, Reno, NV USA; 16 Department of Slavonic Languages and Caucasus Studies, Friedrich-Schiller-Universität Jena, Jena, Germany; 17 Cognitive Science, Department of Humanities, Social and Political Sciences, ETH Zürich, Zürich, Switzerland; 18 Cognitive Science and Psycholinguistics Lab, Central Department of Linguistics, Tribhuvan University, Kathmandu, Nepal; 19 Centre for Neural and Cognitive Sciences, School of Medical Sciences, University of Hyderabad, Hyderabad, India; 20 Department of Communication and Cognition, TiCC, Tilburg University, Tilburg, the Netherlands; 21 Max Planck Institute for Psycholinguistics, Nijmegen, the Netherlands; 22 Department of Chemistry, University of Liverpool, Liverpool, UK; 23 Department of Computer Science, University of Manchester, Manchester, UK; 24 Laboratory for Perceptual and Cognitive Systems, Faculty of Computing, University of Latvia, Riga, Latvia; 25 Department of German, Friedrich-Schiller-Universität Jena, Jena, Germany; 26 School of Communication and Culture, Aarhus University, Aarhus, Denmark; 27 School of Languages and Translation Studies, University of Turku, Turku, Finland; 28 Institute for the Languages and Cultures of the Baltic, Vilnius University, Vilnius, Lithuania; 29 Centro de Investigación Nebrija en Cognición, Universidad Antonio de Nebrija, Madrid, Spain; 30 Department of Languages and Culture, Arctic University of Norway, Tromsø, Norway; 31 Interacting Minds Centre, Aarhus University, Aarhus, Denmark; 32 Centre for Humanities Computing, Department of Culture, Cognition and Computation, Aarhus University, Aarhus, Denmark; 33 Centre of Functionally Integrative Neuroscience, Aarhus University Hospital, Aarhus, Denmark; 34 Department of Computer Engineering, Bogazici University, Istanbul, Turkey

Correction to: *Nature Human Behaviour* 10.1038/s41562-023-01697-4, published online 30 October 2023.

In the version of the article initially published, in Table 1, “Bulgarian” previously read “Bulgaria”, and “Bulgaria” previously read “Eurasia”. These errors have been corrected in the HTML and PDF versions of the article.

