# Supplementary material for: Spatial communication systems across languages reflect universal action constraints
Source: Nat Hum Behav. 2023 Oct 30;7(12):2099–110. doi: 10.1038/s41562-023-01697-4 (PMC10730392; doi:10.1038/s41562-023-01697-4)
Supplement: Supplementary file 1 — Supplementary Sections 1–7, Figs. 1 and 2, and Tables 1–79. [file 41562_2023_1697_MOESM1_ESM.pdf]

nature human behaviour

Article

https://doi.org/10.1038/s41562-023-01697-4

Spatial communication systems across languages reflect universal action constraints

In the format provided by the authors and unedited

Table of Contents

Table of Contents *1*

Supplementary Text *6*

1. Demonstratives Systems for Individual Languages and Term Selection **6**
Arabic (Tunisian) 6
Basque 6
Bulgarian 6
Cantonese 6
Castilian (Spanish) 7
Catalan 7
Danish 7
Dutch 7
English 7
Estonian 8
Finnish 8
Georgian 8
German 8
Italian 8
Japanese 9
Korean 9
Latvian 9
Lithuanian 9
Maltese 10
Mandarin 10
Marathi 10
Nepali 10
Norwegian 10
Telugu 11
Tseltal 11
Turkish 11
Vietnamese 11
Võro 11
Yucatec 12

2. Participants and background information **12**

3. Secondary Data Analyses **12**

4. Supplementary Results **13**

4.1 Main Analyses – Additional Information **13**
4.1.1 Interrogating the main effect of distance 13
4.1.2 Intersubject variability 14

4.2 Main Individual Language Analyses **14**
Arabic (Tunisian)* 14
Basque* 14
Bulgarian* 14
Cantonese 15
Castilian* 15
Catalan* 15
Danish 15
Dutch 15
English 15
Estonian 15
Finnish 16

Georgian* 16
German* 16
Italian 16
Japanese 16
Korean* 17
Latvian 17
Lithuanian 17
Maltese 17
Mandarin 17
Marathi 17
Nepali 17
Norwegian 18
Telugu 18
Tseltal 18
Turkish 18
Vietnamese 18
Võro 18
Yucatec* 18

4.3 A priori models in which separation occurred due to 'no-events': zero values. 18

Arabic (Tunisian) 18
Basque 19
Bulgarian 19
Castilian 19
Catalan 19
Georgian 19
German 20
Korean 20
Telugu 20
Yucatec 20

4.2 Supplementary Figures 21

Supplementary Figure 1. A schematic representation of the experimental setup. The Speaker (participant) is seated at a table, with the Addressee either sitting side-by-side, or opposite the speaker. Color-coded locations mark distance increases of 25cm each. In the schematic, three conceptual regions are represented, such that Region 1 and Region 2, are just as far from the speaker as respectively Region 3 and Region 2 are from the Addressee, when seated opposite. 21
Supplementary Figure 2. Normalized proximal term use (collapsed across the 29 languages; red bars) plotted beside normalized (non-linguistic) object-location memory data from (32) (with/without explicit verbal interference; green/blue bars; see 32). 22

5. Supplementary Tables 23

Supplementary Table 1. Participant demographic information per language. 23
Supplementary Table 2. Cross-tabulation table of the frequency of demonstrative use per language, by addressee position and region. 24
Supplementary Table 3. Random effects in overall GLMM (multilevel model) 26
Supplementary Table 4. Random effects in the GLMMs run per model, showing significant inter-subject variability in all individual languages. 27
Supplementary Table 5. Classification table for the follow-up Tunisian Arabic model, ... 28
overall percentage correct: 90.3% 28
Supplementary Table 6. Fixed coefficients fo00r the follow-up Tunisian Arabic GLMM (multilevel model) (Excluding Region 1). 29
Supplementary Table 7. Classification table for the follow-up Basque GLMM (multilevel model), ... 30
overall percentage correct: 85.7% 30
Supplementary Table 8. Fixed coefficients for the follow-up Basque GLMM (multilevel model) (Excluding Region 1). 31
Supplementary Table 9. Classification table for the follow-up Bulgarian model, overall percentage correct: 96.8% 32

Supplementary Table 10. Fixed coefficients for the follow-up Bulgarian GLMM (multilevel model) (Excluding Region 1). 33
Supplementary Table 11. Classification table for the *a priori* Cantonese model, ... 34
overall percentage correct: 91.4% 34
Supplementary Table 12. Fixed coefficients for the *a priori* Cantonese GLMM (multilevel model). 35
Supplementary Table 13. Classification table for the follow-up Castilian model, ... 36
overall percentage correct: 94.4% 36
Supplementary Table 14. Fixed coefficients for the follow-up Castilian GLMM (multilevel model) (Excluding Region 1 and Proximal term). 37
Supplementary Table 15. Classification table for the follow-up Catalan model, ... 38
overall percentage correct: 91.4% 38
Supplementary Table 16. Fixed coefficients for the follow-up Catalan GLMM (multilevel model) (Excluding Region 1). 39
Supplementary Table 17. Classification table for the *a priori* Danish model, ... 40
overall percentage correct: 85.5% 40
Supplementary Table 18. Fixed coefficients for the *a priori* Danish GLMM (multilevel model). 41
Supplementary Table 19. Classification table for the *a priori* Dutch model, ... 42
overall percentage correct: 84.4% 42
Supplementary Table 20. Fixed coefficients for the *a priori* Dutch GLMM (multilevel model). 43
Supplementary Table 21. Classification table for the *a priori* English model, ... 44
overall percentage correct: 83.2% 44
Supplementary Table 22. Fixed coefficients for the *a priori* English GLMM (multilevel model). 45
Supplementary Table 23. Classification table for the *a priori* Estonian model, ... 46
overall percentage correct: 79.7% 46
Supplementary Table 24. Fixed coefficients for the *a priori* Estonian GLMM (multilevel model). 47
Supplementary Table 25. Classification table for the *a priori* Finnish GLMM (multilevel model), ... 48
overall percentage correct: 76.3% 48
Supplementary Table 26. Fixed coefficients for the *a priori* Finnish GLMM (multilevel model). 49
Supplementary Table 27. Classification table for the follow-up Georgian model, ... 50
overall percentage correct: 86.5% 50
Supplementary Table 28. Fixed coefficients for the follow-up Georgian GLMM (multilevel model) (Excluding Region 1). 51
Supplementary Table 29. Classification table for the follow-up German model, ... 52
overall percentage correct: 81.8% 52
Supplementary Table 30. Fixed coefficients for the follow-up German GLMM (multilevel model) (Excluding proximal term). 53
Supplementary Table 31. Classification table for the *a priori* Italian model, ... 54
overall percentage correct: 95.7% 54
Supplementary Table 32. Fixed coefficients for the *a priori* Italian GLMM (multilevel model). 55
Supplementary Table 33. Classification table for the *a priori* Japanese model, ... 56
overall percentage correct: 88.6% 56
Supplementary Table 34. Fixed coefficients for the *a priori* Japanese GLMM (multilevel model). 57
Supplementary Table 35. Classification table for the follow-up Korean model, ... 58
overall percentage correct: 87.0% 58
Supplementary Table 36. Fixed coefficients for the follow-up Korean GLMM (multilevel model) (Excluding Region 1). 59
Supplementary Table 37. Classification table for the *a priori* Latvian model, ... 60
overall percentage correct: 88.9% 60
Supplementary Table 38. Fixed coefficients for the *a priori* Latvian GLMM (multilevel model). 61
Supplementary Table 39. Classification table for the *a priori* Lithuanian model, ... 62
overall percentage correct: 85.1% 62
Supplementary Table 40. Fixed coefficients for the *a priori* Lithuanian GLMM (multilevel model). 63
Supplementary Table *41*. Classification table for the *a priori* Maltese model, ... 64
overall percentage correct: 94.4% 64
Supplementary Table *42*. Fixed coefficients for the *a priori* Maltese GLMM (multilevel model). 65
Supplementary Table 43. Classification table for the *a priori* Mandarin model, ... 66
overall percentage correct: 91.3% 66

Supplementary Table 44. Fixed coefficients for the *a priori* Mandarin GLMM (multilevel model)........... 67
Supplementary Table 45. Classification table for the *a priori* Marathi model,.. 68
overall percentage correct: 86.5% 68
Supplementary Table 46. Fixed coefficients for the *a priori* Marathi GLMM (multilevel model). 69
Supplementary Table 47. Classification table for the *a priori* Nepali model, overall percentage correct: 92.1% 70
Supplementary Table 48. Fixed coefficients for the *a priori* Nepali GLMM (multilevel model). 71
Supplementary Table 49. Classification table for the *a priori* Norwegian model,....................................... 72
overall percentage correct: 95.3% 72
Supplementary Table 50. Fixed coefficients for the *a priori* Norwegian GLMM (multilevel model)......... 73
Supplementary Table 51. Classification table for the *a priori* Tseltal model, ... 74
overall percentage correct: 86.1% 74
Supplementary Table 52. Fixed coefficients for the *a priori* Tseltal GLMM (multilevel model). 75
Supplementary Table 53. Classification table for the *a priori* Turkish model,... 76
overall percentage correct: 85.3% 76
Supplementary Table 54. Fixed coefficients for the a priori Turkish GLMM (multilevel model)............... 77
Supplementary Table 55. Classification table for the *a priori* Vietnamese model, ... 78
overall percentage correct: 84.5% 78
Supplementary Table 56. Fixed coefficients for the *a priori* Vietnamese GLMM (multilevel model). 79
Supplementary Table 57. Classification table for the *a priori* Võro model,.. 80
overall percentage correct: 87.0% 80
Supplementary Table 58. Fixed coefficients for the *a priori* Võro GLMM (multilevel model)................... 81
Supplementary Table 59. Classification table for the follow-up Yucatec model,....................................... 82
overall percentage correct: 92.2% 82
Supplementary Table 60. Fixed coefficients for the follow-up Yucatec GLMM (multilevel model) (excluding Region 3). 83
Supplementary Table 61. Classification table for the *a priori* Tunisian Arabic model,.............................. 84
overall percentage correct: 93.1% 84
Supplementary Table 62. Fixed coefficients for the *a priori* Tunisian Arabic GLMM (multilevel model)... 85
Supplementary Table 63. Classification table for the *a priori* Basque model,... 86
overall percentage correct: 88.8% 86
Supplementary Table 64. Fixed coefficients for the *a priori* Basque GLMM (multilevel model)............... 87
Supplementary Table 65. Classification table for the *a priori* Bulgarian model, ... 88
overall percentage correct: 97.6% 88
Supplementary Table 66. Fixed coefficients for the *a priori* Bulgarian GLMM (multilevel model). 89
Supplementary Table 67. Classification table for the *a priori* Castilian model,... 90
overall percentage correct: 94.7% 90
Supplementary Table *68*. Fixed coefficients for the *a priori* Castilian GLMM (multilevel model)............. 91
Supplementary Table 69. Classification table for the *a priori* Catalan model, ... 92
overall percentage correct: 91.4% 92
Supplementary Table 70. Fixed coefficients for the *a priori* Catalan GLMM (multilevel model). 93
Supplementary Table 71. Classification table for the *a priori* Georgian model,... 94
overall percentage correct: 90.7% 94
Supplementary Table 72. Fixed coefficients for the *a priori* Georgian GLMM (multilevel model)............ 95
Supplementary Table 73. Classification table for the *a priori* German model,... 96
overall percentage correct: 83.5% 96
Supplementary Table 74. Fixed coefficients for the *a priori* German GLMM (multilevel model). 97
Supplementary Table 75. Classification table for the *a priori* Korean model, ... 98
overall percentage correct: 90.0% 98
Supplementary Table 76. Fixed coefficients for the *a priori* Korean GLMM (multilevel model). 99
Supplementary Table 77. Classification table for the *a priori* Telugu model,.. 100
overall percentage correct: 91.6% 100
Supplementary Table 78. Fixed coefficients for the *a priori* Telugu GLMM (multilevel model). 101
Supplementary Table 79. Classification table for the *a priori* Yucatec model, ... 102
overall percentage correct: 92.2% 102
Supplementary Table 80. Fixed coefficients for the *a priori* Yucatec GLMM (multilevel model)............ 103

6. Open Access Data and Analysis script **104**
7. SI References **105**

Supplementary Text

1. Demonstratives Systems for Individual Languages and Term Selection

Below we briefly present the demonstrative systems for each language tested, together with the usual gloss for specific terms (e.g., proximal, distal). These glosses come with two important caveats. First, they are in most cases not based on empirical evidence, and when they are, the studies concerned are often underpowered (e.g., employing ethnographic methods with only a few participants). Therefore the glosses are subject to testing/confirmation/falsification. Second, for the languages where there is literature discussing the demonstrative system, there is often debate regarding the use of specific terms, and therefore how they are glossed. For each language we label terms ‘3rd term’, ‘4th term’, etc., if there is no consensus in the literature regarding a single gloss. (Notes: Noun position is presented with an ‘N’ in the individual language overviews; if a language is inflected for number and case we give the singular nominative (or absolutive) form in the tables.)

Arabic (Tunisian)

Tunisian Arabic has three demonstratives inflected for number and gender (*S5*). When used as demonstrative pronouns they stand alone as subjects. When they serve as articles, they can appear either before or after the noun.

	PROXIMAL	DISTAL	DISTAL
MASCULINE	hāđa (هذا)	hāka (هاكا)	hāđāka (هذاك)
FEMININE	hāđī (هذي)	hākī (هاكي)	hāđīka (هذيك)

Basque

Basque has three demonstratives inflected for number and case. Adnominal demonstratives have the same forms as demonstrative pronouns and follow the noun. According to Hualde and Ortiz de Urbina (2003; *S2*), the middle term *hori* indicates “proximity to the addressee”, but in other sources *hori* is said to indicate a referent in intermediate distance between ‘proximal’ *hau* and ‘distal’ *hura*.

	PROXIMAL	3RD TERM	DISTAL
NO GENDER	N *hau*	N *hori*	N *hura*

Bulgarian

Bulgarian has proximal and distal demonstratives inflected for gender and number (*S3*). The proximal term serves as the default in non-contrastive uses. Adnominal demonstratives have the same forms as demonstrative pronouns and precede the noun.

	PROXIMAL	DISTAL
MASCULINE	*този/тоя* N	*онзи/оня* N
FEMININE	*тази/тая* N	*онази/оная* N
NEUTER	*това/туй* N	*онова/онуй* N

Cantonese

Cantonese employs the demonstrative forms *nī* ‘this’ and *go* ‘that’, which are generally accompanied by a classifier and optionally by the linking element *go* (as in Mandarin *zhège* and *nàgè*)(*S4*).

	PROXIMAL	DISTAL
NO GENDER	*nī*^{2} *go*^{3} (這個)N	*go*^{2} *go*^{3} (那個) N

Note: Superscript numbers refer to tone.

Castilian (Spanish)

Castilian has three demonstrative forms inflected for gender and number (*S5*). Adnominal demonstratives have the same forms as demonstrative pronouns. There is continued debate regarding whether the system is person-centered, distance-based, or a combination of the two (*34,39, 47, S5*).

	PROXIMAL	3RD TERM	DISTAL
MASCULINE	*este* N	*ese* N	*aquel* N
FEMININE	*esta* N	*esa* N	*aquella* N

Catalan

Catalan has a three-term deictic system (*S6*), similar to Castilian, but in some current varieties of Catalan the three-term contrast has been reduced to a binary opposition. Adnominal demonstratives are inflected for gender and number. They have the same forms as demonstrative pronouns.

	PROXIMAL	3RD TERM	DISTAL
MASCULINE	*aquest* N	*aqueix* N	*aquell* N
FEMININE	*aquesta* N	*aqueixa* N	*aquella* N

Danish

Danish has two sets of demonstratives: The archaic simple demonstratives *denne/dette* 'this', almost exclusively used in written language, and *hin*, 'that' which has fallen out of use, plus the spatially unmarked pronominal *den/det*. In spoken language, the complex demonstratives *den/det her* 'this' and *den/det der* 'that' are used (*S7*). The latter consist of the unmarked simple demonstratives *den/det* and the adverbial demonstratives *her/der* 'here/there'. The unmarked demonstratives indicate gender and number and precede the noun they modify, while the adverbial part may either precede or succeed the noun. In the experiment, participants used the complex demonstratives.

	PROXIMAL	DISTAL
COMMON GENDER	*denne / den her* N	*den / den der* N
NEUTER	*dette / det her* N	*det / det der* N

Dutch

Dutch has proximal and distal demonstratives inflected for gender and number (*S8*). Adnominal demonstratives precede the noun and have the same forms as demonstrative pronouns.

	PROXIMAL	DISTAL
COMMON GENDER	*deze* N	*die* N
NEUTER	*dit* N	*dat* N

English

The English demonstratives are inflected for number and precede the noun they modify (*S9*). Adnominal demonstratives can also be used as independent pronouns but are combined with the dummy pronoun *one* in many contexts.

	PROXIMAL	DISTAL
NO GENDER	*this* N	*that* N

Estonian

Different varieties of Estonian have different deictic systems (*S10*). In the variety tested in this study, speakers use a two-way deictic system with proximal *see* and distal *too*. Demonstratives are inflected for number and case and precede the noun they modify.

	PROXIMAL	DISTAL
NO GENDER	*see* N	*too* N

Finnish

Finnish has two demonstrative forms inflected for number and case. Adnominal demonstratives precede the noun they modify and have the same forms as demonstrative pronouns. Some older studies mention a second distal term (i.e. *se*), but a number of recent studies have shown that *se* serves primarily as a definiteness marker (similar to English *the*) (*S11*).

	PROXIMAL	DISTAL	3RD TERM
NO GENDER	*tämä* N	*tuo* N	<u>se N</u>

Georgian

Georgian has three demonstratives that are inflected for number and case when used as independent pronouns (*S12*). Adnominal demonstratives do not indicate number and distinguish only two cases, i.e. nominative and non-nominative case. Demonstratives precede the noun they modify.

	NEAR SPEAKER	NEAR ADDRESSEE	DISTAL
NO GENDER	*es* (ეს) N	*eg* (ეგ) N	*is* (ის) N

German

There is an archaic distinction between proximal *dieser* 'this.MASC' and distal *jener* 'that.MASC', but the distal form *jener* is no longer used with exophoric reference (*16*). Instead, *dieser* and the stressed forms of *der/die/das* are commonly combined with the adverbial demonstratives *hier, da* and *dort* (to indicate distance). There is a clear contrast between proximal *hier* and distal *da/dort*, but the semantic difference between *da* and *dort* is not fully understood (*dort* is more formal than *da).* To explore the full extent of the German system, we allowed participants to use *hier*, *da*, and *dort*, and therefore we treated German as a 3-term demonstrative system in our analysis.

	PROXIMAL	DISTAL	DISTAL
MASCULINE	*der* N *hier*	*der* N *da*	*der* N *dort*
FEMININE	*die* N *hier*	*die* N *da*	*die* N *dort*
NEUTER	*das* N *hier*	*das* N *da*	*das* N *dort*

Italian

Italian has two demonstrative forms inflected for number and gender (*S13*). Adnominal demonstratives have the same forms as demonstrative pronouns and precede the noun they modify.

	PROXIMAL	DISTAL
MASCULINE	*questo* N	*quello* N
FEMININE	*questa* N	*quella* N

Japanese

Japanese has three demonstrative determiners that precede the noun they modify. Demonstrative pronouns include the same deictic roots as demonstrative determiners (i.e. *ko*-, *so*-, *a*-), but adnominal and pronominal demonstratives are formally distinguished. There is general consensus that *kono* marks a referent's location near the speaker, while *ano* marks an object distal to the speaker, but there is considerable debate regarding whether *sono* is a medial term or alternatively marks the territory near a hearer (*38*).

	PROXIMAL	3RD TERM	DISTAL
NO GENDER	*kono* (この) N	*sono* (その) N	*ano* (あの) N

Korean

Korean has three deictic particles with a particular term for referents near the addressee: *i* 'near speaker', *geu* 'near addressee', *jeo* 'away from speaker and addressee' (*S14*). As demonstratives, the three particles are generally followed by a nominal expression, either a noun or another classifier, e.g., *Jeu geot* 'that thing'. The latter type of expressions are functionally equivalent to demonstrative pronouns in other languages. *Geu* is frequently used for anaphoric reference and can occur without a co-occurring nominal, but in this use *Geu* functions as a third person pronoun rather than a demonstrative.

	NEAR SPEAKER	NEAR ADDRESSEE	DISTAL
NO GENDER	*i* (geot) (이)N	*geu (geot)* (그)N	*jeo (geot)* (저)N

Latvian

Latvian has two demonstratives inflected for gender, number and case (*S15*) - *tas*/*tā*, *šis*/*šī*, *tāds*/*tāda*, *šāds*/*šāda* - that can be used pronominally or adnominally. Adnominal demonstratives precede the noun they modify and have the same forms as demonstrative pronouns. *Šis/šī, šāds/šāda* are used for referents near the speaker while *tas/tā, tāds/ tāda* – for distant referents. *Šis/šī, tas/ tā* are used to refer to objects while *šāds/ šāda, tāds/tāda* – to the properties of objects. In the present experimental setting and semantic context, the near/distant pairs *šis/ šī* and *tas/ tā* will be tested.

	PROXIMAL	DISTAL
MASCULINE	*šis* N	*tas* N
FEMININE	*šī* N	*tā* N

Lithuanian

Lithuanian has four demonstrative forms inflected for gender, number and case, which precede the noun they modify. The adnominal demonstratives are also used as independent pronouns: *tas* – most frequently, *šis* / *šitas* – less frequently, but *anas* – quite rarely. However, *šis* and *šitas* are semantically treated as one (etymologically *šitas* is a compound based on *šis* and *tas*). Traditionally, there is general consensus that *šis* and *šitas* are used for referents near the speaker and *anas* refers to referents that are far away, but *tas* has been interpreted as distance-neutral. However, more recently it has been suggested that *tas* should be treated as the main distal demonstrative since *anas* is not frequently used any more (*S16*). Given the fact that *šis* and *šitas* are semantically treated as one, we analyzed Lithuanian as a 3-term demonstrative system.

	PROXIMAL	DISTAL TERM	3RD TERM
MASCULINE	*šìs / šìtas* N	*tàs* N	*anàs* N
FEMININE	*šì / šità* N	*tà* N	*anà* N

Maltese

Maltese has proximal and distal demonstratives inflected for gender and number (*S17*). The same demonstratives are used as pronouns and as noun modifiers. Adnominal demonstratives precede the noun they modify.

	PROXIMAL	DISTAL
MASCULINE	*dan* N	*dak* N
FEMININE	*din* N	*dik* N

Mandarin

Mandarin demonstratives consist of the deictic elements *zhè* 'this' and *nà* 'that' and the classifiers *ge* in singular and *xiē* in plural. The distal demonstrative *nà-gè* is frequently used with endophoric reference similar to a definite article. Demonstratives precede the noun they modify and can also be used without a co-occurring noun as independent pronouns (*S18*).

	PROXIMAL	DISTAL
NO GENDER	*zhè-ge* (这个)N	*nà-gè* (那个)N

Marathi

Marathi has two demonstrative forms inflected for gender and number. Adnominal demonstratives precede the noun and can also be used as independent pronouns (*S19*).

	PROXIMAL	DISTAL
MASCULINE	*to* (तो) N	*ha* (हा) N
FEMININE	*ti* (ती) N	*hi* (ही) N
NEUTER	*te* (ते) N	*he* (हे) N

Nepali

Nepali has two demonstratives inflected for number (*S20*). Adnominal demonstratives precede the noun and can also be used as independent pronouns.

	PROXIMAL	DISTAL
NO GENDER	*yo* (यो) N	*tyo* (त्यो) N

Norwegian

Demonstratives are inflected for gender and number and precede the noun they modify. The same demonstratives are used as noun modifiers and independent pronouns. Like Danish, Norwegian has both simple and complex demonstratives. The simple demonstratives occur in Standard Norwegian, but the complex forms are also widely used, especially in spoken registers. According to Vindenes (*S21*), there is no clear-cut distinction between demonstratives, third person pronouns and definite articles. Usually, definiteness is indicated by bound articles on the noun and discourse participants are tracked by third person pronouns, but the demonstratives are also often used as definite markers and anaphors.

	PROXIMAL	DISTAL
COMMON GENDER	*denne / den her* N	*den / den der* N
NEUTER	*dette / det her* N	*det / den der* N

Telugu

There are two invariable demonstratives that precede the noun they modify (*S22*). Unlike adnominal demonstratives, demonstrative pronouns are inflected for gender and number, but include the same deictic roots.

	PROXIMAL	DISTAL
NO GENDER	*ī:*(ఈ) N	*ā: (*ఆ*)* N

Tseltal

The Tseltal has two demonstratives that act as circumclitics (*S23*). The two pieces of the demonstrative surround the phrase they are modifying. There is a two-way distinction in Tseltal, proximal and distal (with a third, far distal, possible in the adverbial form of the demonstrative).

	PROXIMAL	DISTAL
NO GENDER	*in* N *i*	*men* N *e*

Turkish

Turkish has a three-way demonstrative system. The system has been variously described as distance based (proximal, distal, medial) or person-centered. More recently the 3rd term, *şu,* has been proposed as an attention shifting term, used when the addressee is not attentionally engaged (*46*). Adnominal demonstratives are uninflected particles that precede the noun, whereas demonstrative pronouns are inflected for number and case.

	PROXIMAL	DISTAL	3RD TERM
NO GENDER	*bu* N	*o* N	*şu* N

Vietnamese

Most studies assume that Vietnamese has a three-way deictic system, but the semantic and pragmatic uses of the three demonstratives is debated. Bui (2014: 47; *S24*) argues that the distinction between *đấy* (allomorph *đó)* and *kia* does not primarily concern distance but newness: *đấy* indicates a referent that is known to the hearer, whereas *kia* is used for newly introduced referents. In addition, there is a demonstrative for invisible referents (i.e. *nọ*). The non-proximal demonstratives *đấy* (*đó)* and *kia* are also used as independent pronouns, but the proximal term is different. The adnominal demonstratives are invariable particles that follow a noun (which may be accompanied by a classifier). In our study, participants were allowed to use any of 5 demonstratives (Này, Đấy/Đó, kia, plus a distal *nọ*, and a proximal *đây*), in order to explore the full extent of Vietnamese demonstrative use. However, since the frequency of use of *no* (only 3 uses in Region 2; 4 in Region 3) and *đây* (15 uses in Region 1; 1 use in Region 2) were so low, we did not include these in our analysis.

	PROXIMAL	DISTAL	3RD TERM	4TH TERM	5TH TERM
NO GENDER	N *này*	N *đấy (đó)*	N *kia*	N *nọ*	N *đây*

Võro

Võro is closely related to Estonian, but in contrast to Estonian, Võro has three rather than two demonstratives. Traditionally, the medial term *taa* is considered to indicate a referent near the addressee, but more recently Pajusalu (2015: 18; *S25*) has argued that *taa* and *tuu* differ mainly with regard to accessibility. All three demonstratives can be used as noun modifiers and pronouns, but *tuu* is primarily a determiner, whereas *taa* is more frequent as a pronoun. All three demonstratives are inflected for number and case and precede the noun they modify.

	PROXIMAL	NEAR ADDRESSEE	DISTAL
NO GENDER	*sjoo (seo)* N	*taa* N	*tuu* N

Yucatec

The Yucatec demonstratives have unusual syntactic properties as they consist of two discontinuous elements: a determiner (or adverb) that occurs inside the clause and a deictic particle clitic that is attached to the clause-final word (*27*). Therefore, demonstratives are arguably not a lexical category, but as they still form a binary contrast of exophoric reference of proximal vs. non-proximal, this does not affect our analysis. The determiner *le* does not indicate any sense of distance or accessibility, but the clause-final deictic clitic particles are contrastive: the "immediate" particle *=a'* indicates a referent that is easily accessible to the interlocutors, or in the domain of manipulable space, and that is preferred for referents in the speaker's reach or proximity, and the "non-immediate" particle *=o'* indicates a referent that is accessible but without expressing proximity. Crucially, the non-immediate particle serves as a default of sorts, in the sense that it is also commonly used for anaphoric reference and definiteness marking.

It is grammatically sound to combine the proximal and non-proximal terms in a single sentence in reference to the same object. A priori we decided to treat such combinations as a third response option. However, throughout the trials, this option was used only 3 times (2 times in Region 1, 1 time in Region 2), so these trials were excluded from the analysis.

	PROXIMAL	DISTAL
NO GENDER	*le* N … *=a'*	*le* N… *=o'*

2. Participants and background information

All participants were native speakers (L1 speakers from birth) of the languages tested, with normal or corrected to normal vision (self-report). Most of the participants were bilingual, as would be expected given the languages tested. We also administered a bilingualism questionnaire (*S26*) to potentially assess language dominance for applicable languages but given that all participants were L1 speakers from birth we do not report these data here. An equal number of male and female participants (self-reported by participants) were recruited (where possible) across languages.

3. Secondary Data Analyses

For three of languages - Catalan, Estonian, Võro - data were collected using the protocols set out above, but earlier analyses (using different statistical techniques) have been published in two manuscripts (publications required for PhD theses):

Reile, M., Plado, H., Gudde, H. B., & Coventry, K. R. (2020). Demonstratives as spatial deictics or something more? Evidence from Common Estonian and Võro**.** *Folia Linguistica, 54 (1).*

Todisco, E., Guijarro-Fuentes, P., & Coventry, K. R. (2021). Analogical levelling in the Majorcan Catalan demonstrative system. *Probus: International Journal of Latin and Romance Linguistics, 33(1),* 33-56*.*

Therefore, the analyses presented here are (technically) secondary data analyses for the three languages.

4. Supplementary Results

4.1 Main Analyses – Additional Information

For the main analyses the decision as to which term is proximal etc. in each language for the analysis was taken *a priori* based on the linguistic analyses provided above (see also Supplementary Table *2*).

In the main analyses reported in the main manuscript both participants and language were included as random effects, and the results are shown in Supplementary Table *3*.

4.1.1 Interrogating the main effect of distance

The main effect of distance in the main analyses is consistent with a distance function that distinguishes between peripersonal/reachable and extrapersonal/non-reachable space. However, one might argue that the pattern is also consistent with a less specific distance function where participants across all languages merely scale demonstrative choice non-linearly. While one cannot completely rule out that possibility, there are four reasons why a reachable/non-reachable distinction maps most closely onto the data, as follows:

A) Processing of space is not just distance based

Using language to describe object location first requires processing of space to determine where objects are located (and to prepare for action). It has been established that spatial terms do involve the activation of non-linguistic regions of the brain involved in processing space when comprehending spatial prepositions (*S27, S28*) or spatial demonstratives (*S29*). Moreover, it is well established that space is processed not by a single brain system, but in a differentiated manner related to perception and action (see *S30, S31* for recent reviews). Studies from single cell recordings with monkeys (e.g. *S32*, S*33*), imaging studies with humans (e.g. *S34*, S*35*) and studies from patients with specific brain lesions (e.g. *S36*, S*37*, S*38*) have shown a dissociation between peripersonal and extrapersonal space processing. For example, Berti and Frasinetti (*S38*) reported the case of a patient with visual neglect who exhibited neglect on a line bisection task in near but not in far space when using a light pen to perform the task. Critically, when using a stick, performance in far space deteriorated, suggesting that extension of reach extended the body, remapping far space as near space. So there is a growing concensus that processing space involves a qualitative distinction between peripersonal and extrapersonal space.

B) Consistency with studies using the same memory game method that have directly manipulated reachability

Work on the processing of space led directly to examination of reachability and demonstrative use in two studies employing the 'memory game method'. Directly inspired by visual neglect work showing an extension of near space using a tool (*S38*), Coventry et al. (S*34*) manipulated reachability in experiments with English and Spanish speakers (two of the languages in the present sample). Participants pointed at objects placed at different distances in front of them either using their hand or a stick. In both languages there was an extension of the use of the proximal term (*this*, *este*) when pointing with the stick to the region beyond the end of the hand to the end of the stick. Therefore extending reach led to a corresponding extension in the use of the proximal term.

In another study (S*36*), participants pointed at objects at locations varying on both the sagittal and lateral planes using their left or right arms. At critical locations where an object to the left or right of the participant was reachable with only one hand, the hand used to point affected choice of *this* or *that* to describe object location as a function of whether the pointing hand could indeed reach the object or not.

Taken together, these studies show reachability and not mere distance affects demonstrative choice in two of the 26 languages tested – English and Spanish. Given that the data are very similar in these studies compared to the data found in the present study (see below), this bolsters the view that reachability maps onto demonstrative choice.

C) Correlations between linguistic and non-linguistic behaviour in previous studies

In another study Coventry et al. (S*35*) tested participants on a non-linguistic object-location memory task and on the language memory game task using the same object distances and found that performance was correlated between tasks. They argued that, if the use of demonstratives is dependent on processing of space (where a peripersonal/exrapersonal distinction is evident), then

one should find that performance on linguistic and non-linguistic variants of the memory game paradigm should be correlated. This was indeed the case.

D) Similarity between the present data across languages and data from previous studies on non-linguistic tasks

Supplementary Figure *2* shows percentage change in proximal term use in the present data (collapsed across languages) plotted beside normalised non-linguistic memory error data by distance (from S*35*). As can be seen in the Figure, the pattern of change in proximal term use across languages mirrors the pattern of change in non-linguistic object-location memory data for the same distances, implicating a relationship between processing of space and demonstrative use in the present data.

In summary, while one cannot definitely rule out a different/more general non-linear distance function, the mapping between reachability and demonstrative choice and the similarity of findings in the present study to non-linguistic spatial data implicates reachability as the most plausible and parsimonious explanation of the distance effect findings observed across languages.

4.1.2 Intersubject variability

Supplementary Table 4 shows significant inter-participant variability results for all individual languages.

4.2 Main Individual Language Analyses

Arabic (Tunisian)*[1]

As can be seen in Supplementary Table *2* (see also Supplementary Tables *61* and *62*), separation occurred in the *a priori* model of Arabic due to the 'no event' condition: the distance effect was so strong that the proximal demonstrative was used in all but 6 trials in Region 1, and never in Region 3 when addressee was seated opposite the speaker. To run a more appropriate analysis of addressee position and distance across Regions 2 and 3, we ran a follow-up model excluding Region 1 (see Supplementary Tables *5* and *6*). For completeness the *a priori* model is presented in section 4.3.

First, the strong correlation between the proximal demonstrative and Region 1 can be seen in the classification table, which never predicts the use of a proximal demonstrative after the elimination of Region 1. The follow-up model in Arabic still revealed a main effect of Region, $F(2, 808) = 5.237$, $p = .005$, $\eta p2 = .013$. The coefficient table shows that the distal and the 3rd term are respectively 25.5 and 38 times more likely to be used in Region 3 compared to the proximal demonstrative.

Basque*

As can be seen in Supplementary Table *2* (see also Supplementary Tables *63* and *64*), separation occurred in the *a priori* model of Basque due to the 'no event' condition: the distance effect showed a strong correlation between the proximal demonstrative and Region 1, such that the proximal demonstrative was never used in Region 3 while side-by-side. To run a more appropriate analysis of addressee position and distance across Region 2 and 3, we ran a follow-up model excluding Region 1 (see Supplementary Tables *7* and *8*). The *a priori* model is presented in section 4.3.

The strong correlation between the proximal demonstrative and Region 1 is supported by the fact that the proximal demonstrative is never predicted after the elimination of Region 1 (see the classification table). The follow-up model in Basque revealed a main effect of Region, $F(2, 808) = 39.52$, $p < .001$, $\eta p2 = .089$. The classification table shows that that the proximal demonstrative is not expected to be used at all when Region 1 is taken out of the analysis.

Bulgarian*

As can be seen in Supplementary Table *2* (see also Supplementary Tables *65* and *66*), separation occurred in the *a priori* model of Bulgarian due to the 'no event' condition: the distance effect was so strong that the distal demonstrative was never used in Region 1 while opposite (and only in 2 trials in Region 1 overall). To run a more appropriate analysis of addressee position and distance across Region 2 and 3, we ran a follow-up model excluding Region 1 (see Supplementary Tables *9* and 10). The *a priori* model is presented in section 4.3.

---

[1] * marks descriptions of follow-up models, due to separation in the *a priori* model. The *a priori* analyses of these are represented in Section 4.2.

The follow-up model showed both main effects: Addressee position, *F*(1, 500) = 6.81, *p* = .009, $\eta p2$= .013, and Region, *F*(1, 500) = 31.608, *p* < .001, $\eta p2$ = .059, but not the interaction. The Region effect showed that, even without Region 1, there was a significant decrease in the use of the proximal demonstrative between Regions 2 and Region 3, with the Exp(Coefficient) showing the use of distal was 221 times more likely in Region 3 than in Region 2. The main, but weak, effect of addressee position did not come out in the coefficients.

Cantonese

The Cantonese data displayed a main effect of Region, *F*(2, 1290) = 32.366, *p* < .001, $\eta p2$ = .048 (see Supplementary Tables *11* and *12*). The data show significantly decreasing odds of proximal demonstrative use beyond Region 1, while the coefficients show a stronger decline of the odds the further away the object is (stronger coefficient for Region 3 compared to Region 2).

Castilian*

As can be seen in Supplementary Table *2* (see also Supplementary Tables *67* and *68*), separation occurred in the *a priori* model of Castilian due to the 'no event' condition: the distance effect was so strong that the proximal demonstrative was never used in Region 3, and the distal demonstrative was never used in Region 1. To run a more appropriate analysis, we ran a follow-up model excluding Region 1 and the proximal demonstrative (see Supplementary Tables *13* and *14*). The *a priori* model is presented in section 4.3.

The follow-up analysis for Castilian showed a Region effect, *F*(1, 708) = 74.623, *p* < .001, $\eta p2$ = .095, with a clear crossover between the 3rd term and the distal term between Region 2 and Region 3, such that the 3rd term is a clear medial term.

Catalan*

As can be seen in Supplementary Table *3* (see also Supplementary Tables *69* and *70*), separation occurred in the *a priori* model of Catalan, due to the 'no event' condition: the distance effect was so strong that the distal demonstrative was never used in Region 1. To run a more appropriate analysis, we ran a follow-up model excluding Region 1 (see Supplementary Tables *15* and *16*). The *a priori* model is presented in section 4.3.

The follow-up analysis for Catalan showed a Region effect, *F*(2, 856) = 20.633, *p* < .001, $\eta p2$ = .046, driven by the distal demonstrative being used far more requently in Region 3 compared to Region 2.

Danish

The Danish data showed a main effect of Region, *F*(2, 1218) = 43.522, *p* < .001, $\eta p2$ = .067 (see Supplementary Tables *17* and *18*). The data show significantly decreasing odds of proximal demonstrative use beyond Region 1, while the coefficients show a stronger decline of the odds the further away the object is (stronger coefficient for Region 3 compared to Region 2).

Dutch

The Dutch data showed a main effect of Region, *F*(2, 1146) = 29.328, *p* < .001, $\eta p2$ = .049 (see Supplementary Tables *19* and *20*). The data show significantly decreasing odds of proximal demonstrative use beyond Region 1, while the coefficients show a stronger decline of the odds the further away the object is (stronger coefficient for Region 3 compared to Region 2).

English

The English data showed a main effect of Region, *F*(2, 1146) = 29.238, *p* < .001, $\eta p2$ = .049 (see Supplementary Tables *21* and *22*). The data show significantly decreasing odds of proximal demonstrative use beyond Region 1, while the coefficients show a stronger decline of the odds the further away the object is (stronger coefficient for Region 3 compared to Region 2).

Estonian

The Estonian data showed a main effect of Region, *F*(2, 1506) = 25.883, *p* < .001, $\eta p2$ = .033 (see Supplementary Tables *23* and *24*). The data show significantly decreasing odds of proximal demonstrative use beyond Region 1, while the coefficients show a stronger decline of the odds the further away the object is (stronger coefficient for Region 3 compared to Region 2).

Finnish

The Finnish data showed a main effect of Region, *F*(4, 950) = 16.872, *p* < .001, $\eta p2$ = .066, and an interaction between region and addressee position, *F*(4, 950) = 4.724, *p* = .001, $\eta p2$= .02 (see Supplementary Tables *25* and *26*). The data show significantly decreasing odds of proximal demonstrative use beyond Region 1, while the coefficients show a stronger decline of the odds the further away the object is (stronger negative coefficient for Region 3 compared to Region 2). The interaction is driven by a decrease in odds of distal term use in Region 3 when interlocutors are face-to-face, in which the 3rd term is used more often.

Georgian*

As can be seen in Supplementary Table *2* (see also Supplementary Tables *71* and *72*), separation occurred in the *a priori* model of Georgian due to the 'no event' condition: the distance effect was so strong that the proximal demonstrative was used in all but 2 trials in Region 1. To run a more appropriate analysis of addressee position and distance across Region 2 and 3, we ran a follow-up model excluding Region 1 (see Supplementary Tables *27* and *28*). The *a priori* model is presented in section 4.3.

The follow-up Georgian model showed main effects of Addressee position, *F*(2, 400) = 18.339, *p* < .001, $\eta p2$ = .084, Region, *F*(2, 400) = 14.714, *p* < .001, $\eta p2$ = .069, and the interaction, *F*(2, 400) = 4.019, *p* = .019, $\eta p2$ = .02. The coefficients show that the distal demonstrative is 45 times more likely to be used in Region 3, when the addressee is seated opposite and all else remains the same. Looking at the frequency table it can be seen that the distal demonstrative is only used in 5 trials when seated side-by-side and behaves like a person-centred term.

German*

As can be seen in Supplementary Table *3* (see also Supplementary Tables *73* and *74*), separation occurred in the *a priori* model of German due to the 'no event' condition: the distance effect was so strong that the proximal demonstrative was used in only 3 trials in Region 3. To run a more appropriate analysis, we ran a follow-up model excluding the proximal demonstrative (see Supplementary Tables *29* and *30*). The *a priori* model is presented in section 4.3.

The German follow-up analysis showed a main effect of Region, *F*(2, 754) = 11.764, *p* < .001, $\eta p2$ = .03. This analysis shows that there is a small effect of distance on the distal and the 3rd term demonstratives in Region 3.

Italian

The Italian data showed a main effect of Region, *F*(2, 1074) = 73.768, *p* < .001, $\eta p2$ = .121, and an Addressee position*Region interaction, *F*(2, 1074) = 3.613, *p* = .027, $\eta p2$= .007 (see Supplementary Tables *31* and *32*). The frequency data show significantly decreasing proximal demonstrative use beyond Region 1. The coefficient table shows that the use of the proximal demonstrative was higher in Region 2 when the experimenter was seated opposite.

Japanese

The Japanese data showed main effects of Region, *F*(4, 1212) = 24.077, *p* < .001, $\eta p2$ = .074, Addressee position, *F*(2, 1212) = 6.165, *p* = .002, $\eta p2$= .01, and an interaction between distance and addressee position, *F*(4, 1212) = 3.027, *p* = .017, $\eta p2$ = .01 (see Supplementary Tables *33*and *34*). The interactions do not come out in the coefficients, but may be due to the fact that the proximal demonstrative (*kono*, the baseline in these comparisons) is impervious to addressee position changes, whereas the distal (*ano*) and 3rd term (*sono*) are affected by addressee position in Region 2 and 3, showing that the use of the 3rd term increases from 25.49% to 36.43% when the addressee is seated opposite. The data show significantly decreasing odds of proximal demonstrative use beyond Region 1, while the coefficients show a stronger decline of the odds the further away the object is (stronger negative coefficient for Region 3 compared to Region 2 when comparing to the distal term (*ano*) (other way round for the 3rd term (*sono*)). The addressee position effect shows an increase of the odds of the use of the 3rd term compared to the distal term, when interlocutors are seated face-to-face.

Korean*

As can be seen in Supplementary Table *3* (see also Supplementary Tables *75* and *76*), separation occurred in the *a priori* model of Korean due to the 'no event' condition: the distance effect was so strong that the 3rd term demonstrative was never used in Region 1 while speaker and addressee were side-by-side. To run a more appropriate analysis, we ran a follow-up model excluding Region 1 (see Supplementary Tables *35* and *36*). The *a priori* model is presented in section 4.3.

As the distance effect is driven by the difference between Region 1 vs Region 2 and Region 3, taking out Region 1 means the distance effect doesn't come out in the *a posteriori* model (however, the distance effect can be clearly seen in Supplementary Table 2; the proximal demonstrative is used in 93.7% of Region 1 trials, and 91.8% of proximal uses was in Region 1). The Korean follow-up analysis showed a main effect of addressee position, $F(2, 784) = 19.729$, $p < .001$, $\eta p2 = .048$, in which the 3rd term is used 4.5 times more when the addressee is seated opposite compared to side-by-side.

Latvian

The Latvian data (see Supplementary Tables *37* and *38*) showed a main effect of Region, $F(2, 1146) = 20.087$, $p < .001$, $\eta p2 = .034$. The data show significantly decreasing odds of proximal demonstrative use beyond Region 1, while the coefficients show a stronger decline of the odds the further away the object is (stronger coefficient for Region 3 compared to Region 2).

Lithuanian

The Lithuanian data (see Supplementary Tables *39* and *40)* showed a main effect of Region, $F(4, 1140) = 24.182$, $p < .001$, $\eta p2 = .078$, and an interaction between distance and addressee position, $F(4, 1140) = 2.821$, $p = .024$, $\eta p2 = .01$. The interaction is driven by the distal term, which is used more often when the addressee is seated opposite the speaker. Furthermore, the data show significantly decreasing odds of proximal demonstrative use beyond Region 1, while the coefficients show a stronger decline of the odds the further away the object is (stronger coefficient for Region 3 compared to Region 2). The addressee position effect shows differences in odds of proximal versus distal use, showing proximal demonstratives are used more frequently in Region 1 when interlocutors are seated opposite compared to side-by-side, while the distal demonstrative is used more in Region 3 when seated opposite.

Maltese

The Maltese data (see Supplementary Tables *41* and *42*) showed a main effect of Region, $F(2, 1218) = 82.384$, $p < .001$, $\eta p2 = .119$. The data show significantly decreasing odds of proximal demonstrative use beyond Region 1, while the coefficients show a stronger decline of the odds the further away the object is (stronger coefficient for Region 3 compared to Region 2).

Mandarin

The Mandarin data (see Supplementary Tables *43* and *44*) showed a main effect of Region, $F(2, 1218) = 113.005$, $p < .001$, $\eta p2 = .157$, and an interaction between region and addressee position, $F(2, 1218) = 4.057$, $p = .018$, $\eta p2 = .007$. The interaction shows higher odds (1.6 times more likely) for proximal demonstrative use in Region 2 when seated face-to-face ($p = .004$). The frequency table shows decreasing proximal demonstrative use beyond Region 1.

Marathi

The Marathi data (see Supplementary Tables *45* and *46*) showed a main effect of Region, $F(2, 714) = 12.156$, $p < .001$, $\eta p2 = .033$. The data show significantly decreasing odds of proximal demonstrative use beyond Region 1, while the coefficients show a stronger decline of the odds the further away the object is (stronger coefficient for Region 3 compared to Region 2).

Nepali

The Nepali data (see Supplementary Tables *47* and *48*) showed a main effect of Region, $F(2, 1146) = 153.823$, $p < .001$, $\eta p2 = .212$. The data show significantly decreasing odds of proximal demonstrative use beyond Region 1, while the coefficients show a stronger decline of the odds the further away the object is (stronger coefficient for Region 3 compared to Region 2).

Norwegian

The Norwegian data (see Supplementary Tables *49* and *50*) showed a main effect of Region, $F(2, 822) = 40.08$, $p < .001$, $\eta p2 = .089$. The data show significantly decreasing odds of proximal demonstrative use beyond Region 1, while the coefficients show a stronger decline of the odds the further away the object is (stronger coefficient for Region 3 compared to Region 2).

Telugu

As can be seen in Supplementary Table *2* (see also Supplementary Tables *77* and *78*), separation occurred in the *a priori* model of Telugu due to the ‘no event’ conditions: the distance effect was so strong that proximal demonstratives were never used in Region 3, while distal demonstratives were never used in Region 1. This means that a full model cannot be run for Telugu: the data shows almost no variance based on addressee position, while the distance effect is a very strong predictor of demonstrative use. The *a priori* model is presented in section 4.3.

Tseltal

The Tseltal data (see Supplementary Tables *51* and *52*) showed a main effect of Region, $F(2, 641) = 21.495$, $p < .001$, $\eta p2 = .063$. The data show significantly decreasing odds of proximal demonstrative use beyond Region 1.

Turkish

The Turkish data (see Supplementary Tables *53* and *54*) showed a main effect of Region, $F(4, 1212) = 29.14$, $p < .001$, $\eta p2 = .088$. The data show significantly decreasing odds of proximal demonstrative use beyond Region 1, while the coefficients show a stronger decline of the odds the further away the object is (stronger coefficient for Region 3 compared to Region 2).

Vietnamese

Note that 2 of the 5 original Vietnamese demonstrative options were used < 2% of the time, and were eliminated as described in the analysis plan. The Vietnamese data (see Supplementary Tables *55* and *56*) showed a main effect of Region, $F(4, 1081) = 24.24$, $p < .001$, $\eta p2 = .082$, with decreasing odds of proximal demonstrative use beyond Region 1, while the coefficients show a stronger decline of the odds the further away the object is (stronger coefficient for Region 3 compared to Region 2).

Võro

The Võro data (see Supplementary Tables *57* and *58*) showed a main effect of Region, $F(4, 960) = 22.961$, $p < .001$, $\eta p2 = .087$. The data show significantly decreasing odds of proximal demonstrative use beyond Region 1, while the coefficients show a stronger decline of the odds of proximal versus distal the further away the object is (stronger coefficient for Region 3 compared to Region 2).

Yucatec*

As can be seen in Supplementary Table *2* (see also Supplementary Tables *79* and *80*), separation occurred in the *a priori* model of Yucatec due to the ‘no event’ condition: the distance effect was so strong that the proximal demonstrative was never used in Region 3. To run a more appropriate analysis of addressee position and distance across Regions 1 and 2, we ran a follow-up model excluding Region 3 (see Supplementary Tables 59 and 60). The *a priori* model is presented in section 4.3.

The follow-up model in Yucatec showed a main effect of Region, $F(1, 497) = 34.875$, $p < .001$, $\eta p2 = .066$. The coefficients show that the distal demonstrative is 11.48 times more likely to be used in Region 2 compared to the proximal demonstrative.

4.3 A priori models in which separation occurred due to ‘no-events’: zero values.

For completeness, we present the *a priori* planned analyses in this section. These are models run on data including zero-values, which causes separation in the models. This means that the model estimates unrealistic coefficients, and effect sizes will be greatly exaggerated.

Arabic (Tunisian) (*Interpret with caution, separation occurred in the model due to zero values)

The Arabic data showed main effects of Addressee position, $F(2, 1212) = 40.216$, $p < .001$, $\eta p2 = .062$ and Region, $F(4, 1212) = 85.772$, $p < .001$, $\eta p2 = .221$, and a Region x Addressee interaction, $F(4, 1212) = 26.911$, $p < .001$, $\eta p2 = .082$. The interaction shows significant interaction effects of addressee position on the 3rd term, at both Region 2 and 3 ($p < .001$), although it is important to note the ‘0-value’ in the frequency table at the Region 1 by Opposite combination. Furthermore, the data show significantly decreasing odds of proximal demonstrative use beyond Region 1, while the coefficients show a stronger decline of the odds the further away the object is (stronger negative coefficient for Region 3 compared to Region 2).

Basque (*Interpret with caution, separation occurred in the model due to zero values)

The Basque data showed main effects of Addressee position, $F(2, 1212) = 40.727$, $p < .001$, $\eta p2 = .063$, Region, $F(4, 1212) = 163.384$, $p < .001$, $\eta p2 = .35$, and the interaction, $F(4, 1212) = 39.638$, $p < .001$, $\eta p2 = .116$. The coefficients show that the interaction is driven by the use of the 3rd term in both Regions 2 and 3 compared to Region 1 ($p < .001$), in which the 3rd term is used less frequently when the addressee is seated opposite, although it should be noted that there is a 0-value in the Region 1, 3rd term, side-by-side cell in the frequency table. The addressee position effect is driven by the 3rd term as well, which is used significantly less frequently when the addressee is seated opposite the speaker. Furthermore, the data show significantly decreasing odds of proximal demonstrative use beyond Region 1, while the coefficients show a stronger decline of the odds the further away the object is (stronger coefficient for Region 3 compared to Region 2).

Bulgarian (*Interpret with caution, separation occurred in the model due to zero values)

The Bulgarian data showed both main effects: Addressee position, $F(1, 750) = 246.54$, $p < .001$, $\eta p2 = .247$, Region, $F(2, 750) = 99.997$, $p < .001$, $\eta p2 = .211$, and the Position*Region interaction, $F(2, 750) = 136.562$, $p < .001$, $\eta p2 = .267$. It is however important to note the 0-value at the Opposite by Region 1 combination in the frequency table, as each model would struggle with rare events/empty values. The frequency table shows decreasing proximal demonstrative use beyond Region 1.

Castilian (*Interpret with caution, separation occurred in the model due to zero values)

The Castilian data showed a main effect of Region, $F(3, 1068) = 8685.471$, $p < .001$, $\eta p2 = .961$, and an interaction effect, $F(2, 1068) = 55.629$, $p < .001$, $\eta p2 = .094$. The frequency table shows that there is a clear distinction between proximal and distal terms, such that the proximal demonstrative is almost always used in Region 1, but never in Region 3; while the distal is never used in Region 1. Furthermore, the data show significantly decreasing odds of proximal demonstrative use beyond Region 1, while the coefficients show a stronger decline of the odds the further away the object is (stronger coefficient for Region 3 compared to Region 2). The interaction is not supported by the coefficients calculation, where all p values are 1.

Catalan (*Interpret with caution, separation occurred in the model due to zero values)

As can be seen in Table 3, separation occurred in the *a priori* model of Catalan, due to the ‘no event’ condition: the distance effect was so strong that the distal demonstrative was never used in Region 1. Furthermore, there is an almost complete lack of variance based on addressee position in Regions 1 and 3.

The Catalan model showed a main effect of region, $F(4, 1284) = 7373.315$, $p < .001$, $\eta p2 = .958$ and an interaction, $F(4, 1284) = 6.343$, $p < .001$, $\eta p2 = .019$. This is due to separation within the multinomial model arising from the ‘no event’ condition and the subsequent implausible parameter estimates (N.B. the distal demonstrative was never used in Region 1).

Georgian (*Interpret with caution, separation occurred in the model due to zero values)

The Georgian data showed main effects of Addressee position, $F(2, 61) = 56.442$, $p < .001$, $\eta p2 = .649$, Region, $F(4, 600) = 176.929$, $p < .001$, $\eta p2 = .541$, and the interaction, $F(4, 600) = 105.343$, $p < .001$, $\eta p2 = .413$. The coefficients show the interaction is apparent at almost every level, in both Distal term, opposite by Region 2 and 3 ($p < .001$), and with the 3rd term, opposite by Region 2 ($p = .011$). The addressee position effect is driven by a decrease in the use of the 3rd term (from 43.46% when seated side-by-side to 20.26% when seated opposite). Lastly, the data show significantly decreasing

odds of proximal demonstrative use beyond Region 1, while the coefficients show a stronger decline of the odds the further away the object is (stronger coefficient for Region 3 compared to Region 2). Note that this effect does not come out as strongly in the coefficients of the distal term, but as can be seen in the frequency table, the use of the distal demonstrative is a rare event when seated side-by-side.

German (*Interpret with caution, separation occurred in the model due to zero values)

The German data showed main effects of Addressee position, $F(2, 1140) = 120.804$, $p < .001$, $\eta p2 = .153$, Region, $F(4, 1140) = 299.437$, $p < .001$, $\eta p2 = .512$, and the interaction, $F(4, 1140) = 109.961$, $p < .001$, $\eta p2 = .278$. As can be seen in the coefficients table, the interaction is driven by the opposite by Region 3 cell of the design, contrasting with a proximal demonstrative 0-value. The addressee position effect does not come out in the coefficients, but in the frequency table it can be seen that the proximal demonstrative is used more often when the addressee is seated opposite the speaker, mostly taking from the distal demonstrative. Furthermore, the data show significantly decreasing odds of proximal demonstrative use beyond Region 1, while the coefficients show a stronger decline of the odds the further away the object is (stronger coefficient for Region 3 compared to Region 2).

Korean (*Interpret with caution, separation occurred in the model due to zero values)

The Korean data showed main effects of Region, $F(4, 1176) = 120.864$, $p < .001$, $\eta p2 = .171$, Addressee position, $F(2, 1176) = 74.241$, $p < .001$, $\eta p2 = .202$, and the interaction, $F(4, 1176) = 66.993$, $p < .001$, $\eta p2 = .186$. The data show significantly decreasing odds of proximal demonstrative use beyond Region 1. The addressee position effect represents the increased use of the 3rd term when the addressee is seated opposite the speaker, while the interaction shows that this increase happens specifically in Regions 2 and 3, when the addressee is seated opposite. As in Japanese, the addressee position effect occurs between the distal and 3rd term.

Telugu (*Interpret with caution, separation occurred in the model due to zero values)

The Telugu frequency data show such a strong effect of distance, and an almost complete lack of variance based on addressee position, that while the model runs it cannot do so reliably (as evidenced by the high confidence intervals). There a main effect of Region, $F(1, 5) = 57012.579$, $p < .001$, $\eta p2 = 1$. The data show significantly decreasing odds of proximal demonstrative use beyond Region 1, while the coefficients show a stronger decline of the odds the further away the object is (stronger coefficient for Region 3 compared to Region 2).

Yucatec (*Interpret with caution, separation occurred in the model due to zero values)

The Yucatec data showed a main effect of Region, $F(2, 20) = 158519.433$, $p < .001$, $\eta p2 = 1$. Note that the combination of proximal and distal demonstratives was only used in 3 trials, so was not included in the analysis. Furthermore, the distal demonstrative is the clear default for referential use in the experimental setting with a total of 85% of produced demonstratives being distal. Apart from this, the pattern of data in Yucatec mirrors other languages, with proximal used mostly in Region 1, with significantly less proximal use the further the object is placed (stronger coefficient for Region 3 compared to Region 2).

4.2 Supplementary Figures

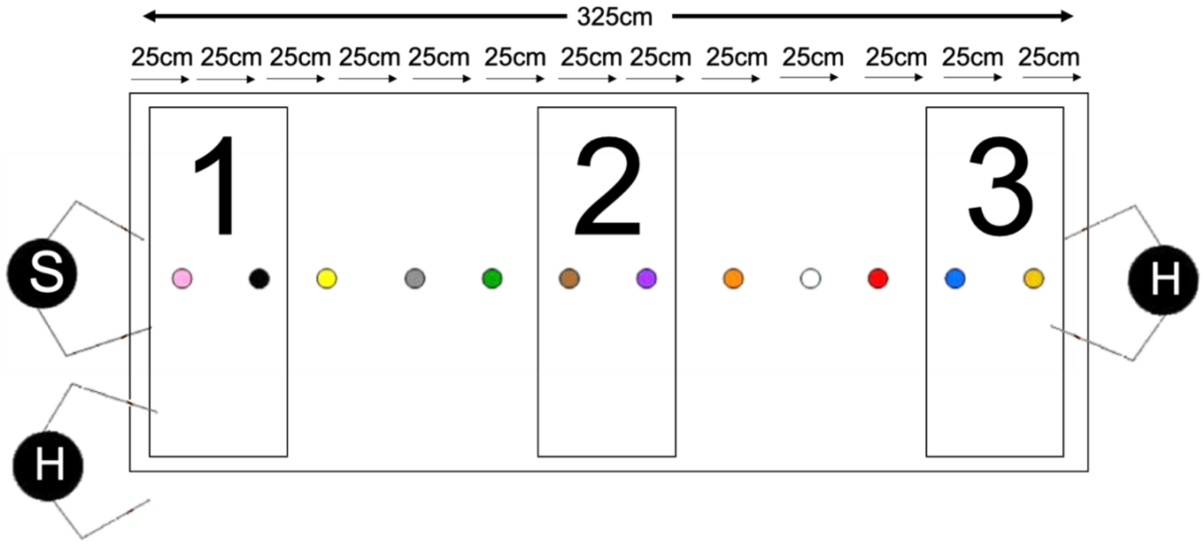

Supplementary Figure 1. A schematic representation of the experimental setup. The Speaker (participant) is seated at a table, with the Addressee either sitting side-by-side, or opposite the speaker. Color-coded locations mark distance increases of 25cm each. In the schematic, three conceptual regions are represented, such that Region 1 and Region 2, are just as far from the speaker as respectively Region 3 and Region 2 are from the Addressee, when seated opposite.

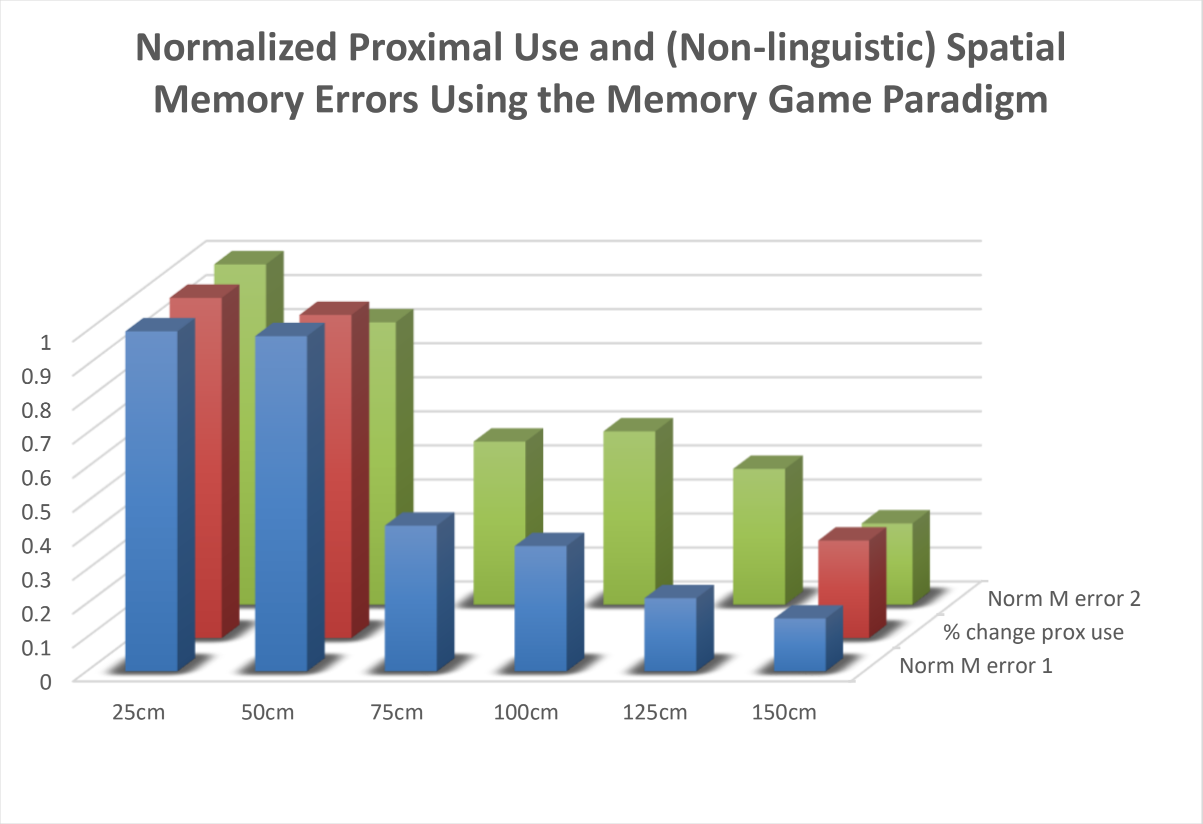

Supplementary Figure 2. Normalized proximal term use (collapsed across the 29 languages; red bars) plotted beside normalized (non-linguistic) object-location memory data from (32) (with/without explicit verbal interference; green/blue bars; see 32).

5. Supplementary Tables

Supplementary Table 1. Participant demographic information per language.

	N (excluded)	Female	Male	Age (M, SD)
Arabic	34	18	16	20.47 (1.42)
Basque	34	23	11	23.35 (7.16)
Bulgarian	21	15	6	35.05 (15.49)
Cantonese	36	17	19	20.69 (2.67)
Castilian	30	18	12	23.5 (5.88)
Catalan	36	25	11	21.86 (3.23)
Danish	34	22	12	23.47 (2.53)
Dutch	33 (1)	16	16	24.61 (3.39)
English	35 (3)	16	16	20.29 (4.6)
Estonian	46 (4)	32	10	27.38 (9.88)
Finnish	27	15	12	Missing data*
Georgian	18 (1)	10	7	27.89 (5.65)
German	42 (10)	16	16	23.19 (4.15)
Italian	30	20	10	29.43 (11.29)
Japanese	37 (3)	17	17	27.35 (7.43)
Korean	36 (3)	16	17	37.78 (10.89)
Latvian	32	16	16	Missing data**
Lithuanian	32	16	16	23.84 (2.4)
Maltese	37 (3)	19	15	26.94 (9.4)
Mandarin	34	18	16	22.27 (2.57)
Marathi	20	6	14	25.55 (3.48)
Nepali	34 (2)	16	16	32.41 (10.17)
Norwegian	23	11	12	23 (2.94)
Telugu	32	14	18	20.72 (1.61)
Tseltal	18	12	6	33.56 (15.89)
Turkish	34	15	19	23.03 (3.96)
Vietnamese	32 (1)	24	8	24.9 (2.41)
Võro	36 (9)	14	13	49.22 (12.7)
Yucatec	21	10	11	34.05 (15.21)

*No age data were collected from this population.
** No specific age data were collected from this population, but all participants were between 19-21.

Supplementary Table 2. Cross-tabulation table of the frequency of demonstrative use per language, by addressee position and region.

		Side-by-side			Opposite		
	**Demon-strative**	**Region 1**	**Region 2**	**Region 3**	**Region 1**	**Region 2**	**Region 3**
**Arabic***							
1st - proximal	Hedhi	200	21	1	202	19	4
2nd - distal	Hedhika	3	160	176	2	155	172
3rd term	Heki	1	23	27	**0**	30	28
**Basque***							
1st - proximal	Hau	189	5	1	191	8	1
2nd - distal	Hori	15	184	97	12	188	116
3rd term	Hura	**0**	15	106	1	8	87
**Bulgarian***							
1st - proximal	този/тоя	124	61	22	126	65	28
2nd - distal	онзи/оня	2	65	104	**0**	61	98
**Cantonese**							
1st - proximal	Nī go	205	63	32	203	77	42
2nd - distal	Go go	11	153	184	13	139	174
**Castilian***							
1st - proximal	Este	170	3	**0**	169	5	**0**
2nd - distal	Aquel	**0**	16	138	**0**	17	143
3rd term	Ese	10	161	42	11	158	37
**Catalan**							
1st - proximal	Aquest	211	74	32	210	87	32
2nd - distal	Aquell	**0**	100	178	**0**	91	178
3rd term	Aqueix	5	42	6	6	38	6
**Danish**							
1st - proximal	Den her	149	50	27	165	51	39
2nd - distal	Den der	55	154	177	39	153	165
**Dutch**							
1st - proximal	Dit/deze	145	45	18	147	37	14
2nd - distal	Dat/die	47	147	174	45	155	178
**English**							
1st - proximal	This	139	33	18	125	42	19
2nd - distal	That	53	159	174	67	150	173
**Estonian**							
1st - proximal	See	208	113	51	210	102	55
2nd - distal	Too	44	139	201	42	150	197
**Finnish**							
1st - proximal	Tämä	96	19	14	90	17	19
2nd - distal	Tuo	26	64	71	35	78	56
3rd term	Se	39	77	74	36	66	85
**Georgian***							
1st - proximal	Es	100	42	26	102	63	23
2nd - distal	Eg	1	3	1	**0**	11	45
3rd - term	Is	1	57	75	**0**	28	34
**German***							
1st - proximal	Hier	180	12	**0**	180	17	3
2nd - distal	Dort	7	70	163	5	70	156
3rd - term	Da	5	110	29	7	105	33
**Italian**							
1st - proximal	Questo	175	12	2	168	17	4
2nd - distal	Quello	5	168	178	12	163	176
**Japanese**							
1st - proximal	Kono	192	4	3	191	4	2
2nd - distal	Ano	3	75	179	3	60	129
3rd term	Sono	9	125	22	10	140	73
**Korean***							
1st - proximal	I-geot	185	5	5	186	12	11
2nd - distal	Jeo-geot	13	177	184	8	154	146
3rd term	Geu-Geot	**0**	16	9	4	32	41
**Latvian**							
1st proximal	Šis/ šī	145	80	59	148	90	66

2nd distal	Tas/tā	47	112	133	44	102	126
**Lithuanian**							
1st proximal	Šis/šitas	160	58	26	175	58	20
2nd distal	Tas	28	123	113	13	126	129
3rd term	Anas	4	11	53	4	8	43
**Maltese**							
1st - proximal	Dan/din	129	34	24	139	39	28
2nd - distal	Dak/dik	75	170	180	65	165	176
**Mandarin**							
1st - proximal	Zhège	195	31	7	184	50	8
2nd - distal	Nàgè	9	173	197	20	154	196
**Marathi**							
1st - proximal	To/ti/te	100	52	38	98	59	42
2nd - distal	Ha/hi/he	20	68	82	22	61	78
**Nepali**							
1st - proximal	Yo	187	37	2	183	37	1
2nd - distal	Tyo	5	155	190	9	155	191
**Norwegian**							
1st - proximal	Den her	40	15	13	42	15	14
2nd - distal	Den der	98	123	125	96	123	124
**Telugu**							
1st - proximal	Ī:	192	77	**0**	192	74	**0**
2nd - distal	Ā:	**0**	115	192	**0**	118	192
**Tseltal**							
1st - proximal	In N i	95	24	20	93	31	11
2nd - distal	Men N e	13	84	87	15	77	97
**Turkish**							
1st - proximal	Bu	197	48	18	197	65	20
2nd - distal	O	5	128	163	1	112	168
3rd - term	Şu	2	28	23	6	27	16
**Vietnamese**							
1st - proximal	Này	168	24	13	165	33	17
2nd - distal	Kia	13	119	143	4	114	130
3rd term	Đấy/Đó	2	40	29	5	38	36
(4th term)	*Đây*	*3*	*1*	***0***	*12*	***0***	***0***
(5th term)	*Nọ*	***0***	*2*	*1*	***0***	*1*	*3*
**Võro**							
1st proximal	Sjoo	110	9	2	128	15	2
2nd distal	Tuu	11	133	153	5	119	152
3rd term	Taa	41	20	7	29	28	8
**Yucatec***							
1st proximal	A'	44	14	**0**	40	12	**0**
2nd distal	O'	82	112	126	84	113	126
(Combination)		***0***	***0***	***0***	*2*	*1*	***0***

*See section 3.1 for a description of 'separation' in multilevel models when zero values occur (bold font).

(italized terms were excluded from analysis as their use was <2%)

Supplementary Table 3. Random effects in overall GLMM (multilevel model)

		Beta	Beta SE	Wald Z	p-value	95% CI
Participants	Distal term	2.003***	0.194	10.307	<.001	[1.656 - 2.423]
	Third term	1.198***	0.22	5.448	<.001	[0.836 - 1.717]
Language	Distal term	1.197***	0.334	3.588	<.001	[0.693 - 2.067]
	Third term	14.357***	4.417	3.251	<.001	[7.856 - 26.237]

**p* <.05; ***p* <.01; ****p*< .001

Supplementary Table 4. Random effects in the GLMMs run per model, showing significant inter-subject variability in all individual languages.

		Estimate	SE	Wald Z	95% CI
Arabic[i]	Distal Term	1.328*	.589	2.255	[.56-3.17]
	Third term	7.798**	2.629	2.966	[4.03-15.10]
Basque[i]	Distal Term	3.271**	1.089	3.003	[1.70-6.28]
	Third term	2.864*	1.153	2.485	[1.30-6.30]
Bulgarian[i]	Distal Term	19.779**	7.453	2.654	[9.45-41.39]
Cantonese	Distal Term	6.206***	1.756	3.534	[3.56-10.81]
Castilian[i]	Distal Term	5.938*	2.327	2.552	[2.76-12.80]
	Third term	4.414**	1.565	2.820	[2.20-8.84]
Catalan[i]	Distal Term	11.240***	3.338	3.367	[6.28-20.12]
	Third term	7.332**	2.350	3.120	[3.91-13.74]
Danish	Distal Term	2.738***	.809	3.383	[1.53-4.89]
Dutch	Distal Term	.899**	.301	2.988	[.47-1.73]
English	Distal Term	.920**	.322	2.857	[.46-1.83]
Estonian	Distal Term	.889***	.253	3.510	[.51-1.55]
Finnish	Distal Term	2.356**	.849	2.776	[1.16-4.77]
	Third term	7.418**	2.484	2.987	[3.85-14.30]
Georgian[i]	Distal Term	3.632*	1.810	2.007	[1.37-9.64]
	Third term	8.284*	3.622	2.287	[3.52-19.52]
German[i]	Distal Term	.416	.239	1.737	[.13-1.29]
	Third term	1.362**	.517	2.638	[.65-2.86]
Italian	Distal Term	1.639**	.594	2.759	[.81-3.33]
Japanese	Distal Term	2.648**	.870	3.045	[1.39-5.04]
	Third term	.495	.320	1.549	[.14-1.76]
Korean[i]	Distal Term	2.274**	.767	2.966	[1.17-4.40]
	Third term	4.594**	1.719	2.673	[2.21-9.57]
Latvian	Distal Term	8.972***	2.715	3.305	[4.96-16.24]
Lithuanian	Distal Term	5.918**	1.814	3.263	[3.25-10.79]
	Third term	5.669**	1.924	2.946	[2.91-11.03]
Maltese	Distal Term	22.927***	6.879	3.333	[12.73-41.28]
Mandarin	Distal Term	1.097**	.367	2.987	[.57-2.11]
Marathi	Distal Term	5.962**	2.281	2.613	[2.82-12.62]
Nepali	Distal Term	2.289**	.814	2.812	[1.14-4.60]
Norwegian	Distal Term	13.386**	4.902	2.730	[6.53-27.44]
Telugu	Distal Term	1.902**	.692	2.750	[.93-3.88]
Tseltal	Distal Term	1.058*	.446	2.372	[.46-2.42]
Turkish	Distal Term	3.026**	.922	3.281	[1.66-5.50]
	Third term	2.074**	.794	2.614	[.98-4.39]
Vietnamese	Distal Term	5.575**	1.782	3.128	[2.98-10.43]
	Third term	5.750**	1.987	2.893	[2.92-11.32]
Võro	Distal Term	3.464**	1.199	2.890	[1.76-6.83]
	Third term	3.405**	1.251	2.723	[1.66-6.99]
Yucatec[i]	Distal Term	5.900*	2.357	2.503	[2.70-12.91]

[i]Indicates the random effects represented are from the *a posteriori* model of a given language, the *a priori* random effects are included in the supplementary information.

Supplementary Table 5. Classification table for the follow-up Tunisian Arabic model, overall percentage correct: 90.3%

		Predicted		
		Proximal	Distal	3rd term
Observed	Proximal	0	43	2
		0.00%	95.60%	4.40%
	Distal	0	653	10
		0.00%	98.50%	1.50%
	3rd term	0	24	84
		0.00%	22.20%	77.80%

Supplementary Table 6. Fixed coefficients fo00r the follow-up Tunisian Arabic GLMM (multilevel model) (Excluding Region 1).

	Coefficient	Std. Error	t-value	p-value	95% Confidence Interval	Exp(Coefficient)	95% Confidence Interval for Exp(Coefficient)
Distal term: Intercept	2.174***	0.401	5.422	<.001	(1.383, 2.964)	8.79	(3.987, 19.379)
Position opposite	0.067	0.447	0.151	.880	(-0.81, 0.945)	1.07	(0.445, 2.572)
Region 3	3.241**	1.139	2.846	.005	(1.006, 5.476)	25.557	(2.733, 238.967)
Opp*Region 3	-1.495	1.067	-1.402	.161	(-3.589, 0.598)	0.224	(0.028, 1.819)
3rd term: Intercept	-1.923*	0.759	-2.535	.013	(-3.434, -0.413)	0.146	(0.032, 0.662)
Position opposite	0.954	0.693	1.378	.169	(-0.405, 2.313)	2.596	(0.667, 10.109)
Region 3	3.638**	1.144	3.18	.002	(1.392, 5.883)	38.007	(4.024, 358.961)
Opp*Region 3	-2.236	1.186	-1.885	.060	(-4.565, 0.093)	0.107	(0.01, 1.097)

Reference categories are as follows: Demonstrative: proximal term, Position of addressee: side-by-side; Region: Region 2;
*=p <.05; ** = p <.01; *** = p< .001

Supplementary Table 7. Classification table for the follow-up Basque GLMM (multilevel model), overall percentage correct: 85.7%

		Predicted		
		Proximal	Distal	3rd term
Observed	Proximal	0	15	0
		0.00%	100.00%	0.00%
	Distal	0	528	57
		0.00%	90.30%	9.70%
	3rd term	0	45	171
		0.00%	20.80%	79.20%

Supplementary Table 8. Fixed coefficients for the follow-up Basque GLMM (multilevel model) (Excluding Region 1).

	Coefficient	Std. Error	t-value	p-value	95% Confidence Interval	Exp(Coefficient)	95% Confidence Interval for Exp(Coefficient)
Distal term: Intercept	3.739***	0.585	6.397	<.001	(2.592, 4.887)	42.064	(13.354, 132.502)
Position opposite	-0.451	0.583	-0.774	.439	(-1.595, 0.692)	0.637	(0.203, 1.998)
Region 3	0.895	1.046	0.857	.392	(-1.157, 2.948)	2.449	(0.315, 19.062)
Opp*Region 3	0.655	1.425	0.46	.646	(-2.141, 3.451)	1.926	(0.118, 31.544)
3rd term: Intercept	0.359	0.624	0.576	.565	(-0.87, 1.588)	1.432	(0.419, 4.895)
Position opposite	-1.142	0.782	-1.461	.144	(-2.676, 0.392)	0.319	(0.069, 1.481)
Region 3	4.244***	1.006	4.22	<.001	(2.27, 6.219)	69.705	(9.679, 501.973)
Opp*Region 3	0.753	1.492	0.505	.614	(-2.176, 3.682)	2.124	(0.114, 39.745)

Reference categories are as follows: Demonstrative: proximal term, Position of addressee: side-by-side; Region: Region 2;
*=p <.05; ** = p <.01; *** = p< .001

Supplementary Table 9. Classification table for the follow-up Bulgarian model, overall percentage correct: 96.8%

		Predicted	
		Proximal	Distal
Observed	Proximal	170	6
		96.60%	3.40%
	Distal	10	318
		3.00%	97.00%

Supplementary Table 10. Fixed coefficients for the follow-up Bulgarian GLMM (multilevel model) (Excluding Region 1).

	Coefficient	Std. Error	t-value	p-value	95% Confidence Interval	Exp(Coefficient)	95% Confidence Interval for Exp(Coefficient)
Distal term: Intercept	-0.14	1.199	-0.116	.908	(-2.64, 2.361)	0.87	(0.071, 10.598)
Position opposite	-0.623	0.577	-1.079	.281	(-1.757, 0.511)	0.536	(0.173, 1.667)
Region 3	5.4***	0.994	5.435	<.001	(3.448, 7.352)	221.319	(31.423, 1558.808)
Opp*Region 3	-0.882	0.555	-1.59	.113	(-1.973, 0.208)	0.414	(0.139, 1.232)

Reference categories are as follows: Demonstrative: proximal term, Position of addressee: side-by-side; Region: Region 2;
*=p <.05; ** = p <.01; *** = p< .001

Supplementary Table 11. Classification table for the *a priori* Cantonese model, overall percentage correct: 91.4%

		Predicted	
		Proximal	Distal
Observed	Proximal	552	70
		88.70%	11.30%
	Distal	42	632
		6.20%	93.80%

Supplementary Table 12. Fixed coefficients for the *a priori* Cantonese GLMM (multilevel model).

	Coefficient	Std. Error	t-value	p-value	95% Confidence Interval	Exp(Coefficient)	95% Confidence Interval for Exp(Coefficient)
Distal term: Intercept	-4.498***	0.673	-6.681	<.001	(-5.827, -3.169)	0.011	(0.003, 0.042)
Position opposite	0.304	0.553	0.549	.583	(-0.781, 1.388)	1.355	(0.458, 4.008)
Region							
Region 3	7.545***	0.943	7.999	<.001	(5.695, 9.396)	1891.61	(297.261, 12037.207)
Region 2	5.836***	0.794	7.354	<.001	(4.279, 7.393)	342.34	(72.167, 1623.974)
Interactions:							
Opp × Region 3	-0.981*	0.494	-1.987	.047	(-1.949, -0.013)	0.375	(0.142, 0.988)
Opp × Region 2	-0.844	0.519	-1.625	.104	(-1.863, 0.175)	0.43	(0.155, 1.191)

Reference categories are as follows: Demonstrative: proximal term, Position of addressee: side-by-side; Region: Region 1;
*=p <.05; ** = p <.01; *** = p< .001

Supplementary Table 13. Classification table for the follow-up Castilian model,
overall percentage correct: 94.4%

		Predicted	
		3rd term	Distal
Observed	3rd term	382	16
		96.00%	4.00%
	Distal	24	290
		7.60%	92.40%

Supplementary Table 14. Fixed coefficients for the follow-up Castilian GLMM (multilevel model) (Excluding Region 1 and Proximal term).

	Coefficient	Std. Error	t-value	p-value	95% Confidence Interval	Exp(Coefficient)	95% Confidence Interval for Exp(Coefficient)
Distal term: Intercept	-3.549***	0.65	-5.46	<.001	(-4.848, -2.251)	0.029	(0.008, 0.105)
Position opposite	0.104	0.475	0.218	.827	(-0.828, 1.035)	1.109	(0.437, 2.816)
Region 3	5.351***	0.753	7.106	<.001	(3.872, 6.829)	210.744	(48.052, 924.266)
Opp*Region 3	0.364	0.592	0.614	.539	(-0.799, 1.526)	1.439	(0.45, 4.6)

Reference categories are as follows: Demonstrative: proximal term, Position of addressee: side-by-side; Region: Region 2;
*=p <.05; ** = p <.01; *** = p< .001

Supplementary Table 15. Classification table for the follow-up Catalan model, overall percentage correct: 91.4%

		Predicted		
		Proximal	Distal	3rd term
Observed	Proximal	186	30	9
		82.70%	13.30%	4.00%
	Distal	12	513	22
		2.20%	93.80%	4.00%
	3rd term	0	15	77
		0.00%	16.30%	83.70%

Supplementary Table 16. Fixed coefficients for the follow-up Catalan GLMM (multilevel model) (Excluding Region 1).

	Coefficient	Std. Error	t-value	p-value	95% Confidence Interval	Exp(Coefficient)	95% Confidence Interval for Exp(Coefficient)
Distal term:							
Intercept	0.253	0.663	0.381	.705	(-1.09, 1.596)	1.288	(0.336, 4.932)
Position opposite	-0.586*	0.279	-2.102	.036	(-1.133, -0.039)	0.557	(0.322, 0.962)
Region							
Region 3	3.77***	0.655	5.76	<.001	(2.486, 5.055)	43.384	(12.007, 156.752)
Interaction:							
SBS × Region 3	0.586	0.398	1.472	.141	(-0.195, 1.367)	1.796	(0.823, 3.922)
3rd term:							
Intercept	-1.677*	0.769	-2.181	.034	(-3.219, -0.135)	0.187	(0.04, 0.873)
Position opposite	-0.68	0.526	-1.293	.196	(-1.713, 0.352)	0.507	(0.18, 1.423)
Region							
Region 3	0.191	1.576	0.121	.903	(-2.902, 3.284)	1.211	(0.055, 26.685)
Interaction:							
SBS × Region 3	0.68	0.621	1.095	.274	(-0.538, 1.899)	1.974	(0.584, 6.676)

Reference categories are as follows: Demonstrative: proximal term, Position of addressee: side-by-side; Region: Region 2;
*=p <.05; ** = p <.01; *** = p< .001

Supplementary Table 17. Classification table for the *a priori* Danish model, overall percentage correct: 85.5%

		Predicted	
		Proximal	Distal
Observed	Proximal	377	104
		78.40%	21.60%
	Distal	73	670
		9.80%	90.20%

Supplementary Table 18. Fixed coefficients for the *a priori* Danish GLMM (multilevel model).

	Coefficient	Std. Error	t-value	p-value	95% Confidence Interval	Exp(Coefficient)	95% Confidence Interval for Exp(Coefficient)
Distal term: Intercept	-1.354***	0.351	-3.856	<.001	(-2.058, -0.65)	0.258	(0.128, 0.522)
Position opposite	-0.577	0.309	-1.867	.062	(-1.183, 0.029)	0.562	(0.306, 1.03)
Region							
Region 3	3.899***	0.494	7.889	<.001	(2.929, 4.869)	49.357	(18.717, 130.155)
Region 2	2.795***	0.393	7.117	<.001	(2.024, 3.565)	16.361	(7.572, 35.353)
Interactions:							
Opp × Region 3	-0.073	0.399	-0.183	.855	(-0.856, 0.709)	0.93	(0.425, 2.033)
Opp × Region 2	0.54	0.381	1.418	.157	(-0.207, 1.287)	1.716	(0.813, 3.623)

Reference categories are as follows: Demonstrative: proximal term, Position of addressee: side-by-side; Region: Region 1;
*=p <.05; ** = p <.01; *** = p< .001

Supplementary Table 19. Classification table for the *a priori* Dutch model,
overall percentage correct: 84.4%

		Predicted	
		Proximal	Distal
Observed	Proximal	299	107
		73.60%	26.40%
	Distal	73	673
		9.80%	90.20%

Supplementary Table 20. Fixed coefficients for the *a priori* Dutch GLMM (multilevel model).

	Coefficient	Std. Error	t-value	p-value	95% Confidence Interval	Exp(Coefficient)	95% Confidence Interval for Exp(Coefficient)
Distal term: Intercept	-1.273***	0.321	-3.966	<.001	(-1.905, -0.641)	0.28	(0.149, 0.527)
Position opposite	-0.064	0.244	-0.265	.791	(-0.542, 0.413)	0.938	(0.581, 1.512)
Region							
Region 3	3.801***	0.577	6.584	<.001	(2.669, 4.934)	44.764	(14.419, 138.974)
Region 2	2.614***	0.441	5.931	<.001	(1.749, 3.479)	13.657	(5.751, 32.431)
Interactions:							
Opp × Region 3	0.357	0.376	0.95	.342	(-0.381, 1.095)	1.429	(0.683, 2.989)
Opp × Region 2	0.34	0.339	1.003	.316	(-0.325, 1.006)	1.406	(0.722, 2.735)

Reference categories are as follows: Demonstrative: proximal term, Position of addressee: side-by-side; Region: Region 1;
*=p <.05; ** = p <.01; *** = p< .001

Supplementary Table 21. Classification table for the *a priori* English model, overall percentage correct: 83.2%

		Predicted	
		Proximal	Distal
Observed	Proximal	256	120
		68.10%	31.90%
	Distal	74	702
		9.50%	90.50%

Supplementary Table 22. Fixed coefficients for the *a priori* English GLMM (multilevel model).

	Coefficient	Std. Error	t-value	p-value	95% Confidence Interval	Exp(Coefficient)	95% Confidence Interval for Exp(Coefficient)
Distal term: Intercept	-1.074**	0.306	-3.515	<.001	(-1.677, -0.471)	0.342	(0.187, 0.625)
Position opposite	0.4	0.236	1.699	.089	(-0.062, 0.862)	1.492	(0.94, 2.368)
Region							
Region 3	3.571***	0.486	7.342	<.001	(2.617, 4.526)	35.568	(13.695, 92.374)
Region 2	2.844***	0.378	7.529	<.001	(2.103, 3.585)	17.189	(8.191, 36.071)
Interactions:							
Opp × Region 3	-0.462	0.556	-0.831	.406	(-1.554, 0.629)	0.63	(0.212, 1.877)
Opp × Region 2	-0.719	0.391	-1.838	.066	(-1.486, 0.049)	0.487	(0.226, 1.05)

Reference categories are as follows: Demonstrative: proximal term, Position of addressee: side-by-side; Region: Region 1;
*=p <.05; ** = p <.01; *** = p< .001

Supplementary Table 23. Classification table for the *a priori* Estonian model, overall percentage correct: 79.7%

		Predicted	
		Proximal	Distal
Observed	Proximal	567	172
		76.70%	23.30%
	Distal	135	638
		17.50%	82.50%

Supplementary Table 24. Fixed coefficients for the *a priori* Estonian GLMM (multilevel model).

	Coefficient	Std. Error	t-value	p-value	95% Confidence Interval	Exp(Coefficient)	95% Confidence Interval for Exp(Coefficient)
Distal term: Intercept	-1.757***	0.332	-5.285	<.001	(-2.409, -1.104)	0.173	(0.09, 0.332)
Position opposite	-0.064	0.305	-0.209	.834	(-0.662, 0.535)	0.938	(0.516, 1.707)
Region							
Region 3	3.318***	0.514	6.457	<.001	(2.31, 4.326)	27.6	(10.073, 75.623)
Region 2	2.02***	0.425	4.752	<.001	(1.186, 2.854)	7.541	(3.276, 17.363)
Interactions:							
Opp × Region 3	-0.04	0.36	-0.111	.911	(-0.746, 0.666)	0.961	(0.474, 1.946)
Opp × Region 2	0.267	0.382	0.698	.485	(-0.483, 1.017)	1.306	(0.617, 2.764)

Reference categories are as follows: Demonstrative: proximal term, Position of addressee: side-by-side; Region: Region 1;
*=p <.05; ** = p <.01; *** = p< .001

Supplementary Table 25. Classification table for the *a priori* Finnish GLMM (multilevel model), overall percentage correct: 76.3%

		Predicted		
		Proximal	Distal	3rd term
Observed	Proximal	205	39	11
		80.40%	15.30%	4.30%
	Distal	46	241	43
		13.90%	73.00%	13.00%
	3rd term	29	60	288
		7.70%	15.90%	76.40%

Supplementary Table 26. Fixed coefficients for the *a priori* Finnish GLMM (multilevel model).

	Coefficient	Std. Error	t-value	p-value	95% Confidence Interval	Exp(Coefficient)	95% Confidence Interval for Exp(Coefficient)
Distal term:							
Intercept	-1.763***	0.444	-3.97	<.001	(-2.649, -0.877)	0.172	(0.071, 0.416)
Position opposite	0.417	0.538	0.776	.438	(-0.639, 1.474)	1.518	(0.528, 4.365)
Region							
Region 3	3.734***	0.674	5.537	<.001	(2.41, 5.057)	41.832	(11.137, 157.128)
Region 2	3.136***	0.585	5.356	<.001	(1.987, 4.285)	23.005	(7.293, 72.573)
Interactions:							
Opp × Region 3	-1.187*	0.472	-2.515	.012	(-2.114, -0.261)	0.305	(0.121, 0.77)
Opp × Region 2	0.001	0.58	0.002	.999	(-1.138, 1.14)	1.001	(0.32, 3.127)
3rd term:							
Intercept	-1.844**	0.655	-2.815	.007	(-3.165, -0.523)	0.158	(0.042, 0.593)
Position opposite	-0.087	0.564	-0.154	.878	(-1.194, 1.02)	0.917	(0.303, 2.773)
Region							
Region 3	3.981***	0.661	6.024	<.001	(2.684, 5.277)	53.549	(14.64, 195.864)
Region 2	3.588***	0.516	6.953	<.001	(2.575, 4.6)	36.149	(13.133, 99.506)
Interactions:							
Opp × Region 3	-0.026	0.494	-0.053	.958	(-0.995, 0.943)	0.974	(0.37, 2.568)
Opp × Region 2	-0.156	0.569	-0.274	.784	(-1.273, 0.961)	0.856	(0.28, 2.614)

Reference categories are as follows: Demonstrative: proximal term, Position of addressee: side-by-side; Region: Region 1;
*=p <.05; ** = p <.01; *** = p< .001

Supplementary Table 27. Classification table for the follow-up Georgian model, overall percentage correct: 86.5%

		Predicted		
		Proximal	Distal	3rd term
Observed	Proximal	130	6	18
		84.40%	3.90%	11.70%
	Distal	9	50	1
		15.00%	83.30%	1.70%
	3rd term	17	4	173
		8.80%	2.10%	89.20%

Supplementary Table 28. Fixed coefficients for the follow-up Georgian GLMM (multilevel model) (Excluding Region 1).

	Coefficient	Std. Error	t-value	p-value	95% Confidence Interval	Exp(Coefficient)	95% Confidence Interval for Exp(Coefficient)
Distal term: Intercept	-3.656***	0.727	-5.026	<.001	(-5.151, -2.161)	0.026	(0.006, 0.115)
Position opposite	0.771	0.902	0.855	.939	(-1.003, 2.545)	2.162	(0.367, 12.745)
Region 3	-0.117	1.411	-0.083	.934	(-2.891, 2.656)	0.889	(0.056, 14.243)
Opp*Region 3	3.799**	1.397	2.72	.007	(1.053, 6.544)	44.643	(2.867, 695.162)
3rd term: Intercept	0.481	0.679	0.709	.494	(-1.028, 1.991)	1.618	(0.358, 7.321)
Position opposite	-2.19**	0.632	-3.468	<.001	(-3.432, -0.948)	0.112	(0.032, 0.387)
Region 3	1.6***	0.366	4.376	<.001	(0.881, 2.319)	4.954	(2.414, 10.165)
Opp*Region 3	0.322	0.732	0.44	.660	(-1.117, 1.762)	1.38	(0.327, 5.823)

Reference categories are as follows: Demonstrative: proximal term, Position of addressee: side-by-side; Region: Region 2;
*=p <.05; ** = p <.01; *** = p< .001

Supplementary Table 29. Classification table for the follow-up German model, overall percentage correct: 81.8%

		Predicted	
		Distal	3rd term
Observed	Distal	407	64
		86.4%	13.6%
	3rd term	74	215
		25.6%	74.4%

Supplementary Table 30. Fixed coefficients for the follow-up German GLMM (multilevel model) (Excluding proximal term).

	Coefficient	Std. Error	t-value	p-value	95% Confidence Interval	Exp(Coefficient)	95% Confidence Interval for Exp(Coefficient)
3rd term: Intercept	0.009	0.729	0.013	.990	(-1.422, 1.441)	1.01	(0.241, 4.224)
Position opposite	-1.063	0.872	-1.22	.223	(-2.774, 0.647)	0.345	(0.062, 1.91)
Regions:							
Region 3	2.061*	0.868	2.375	.018	(0.357, 3.764)	7.851	(1.429, 43.128)
Region 2	-0.425	0.706	-0.602	.547	(-1.811, 0.961)	0.654	(0.163, 2.615)
Interactions:							
Opp*Region 3	0.891	0.972	0.917	.359	(-1.016, 2.798)	2.437	(0.362, 16.413)
Opp*Region 2	1.158	0.922	1.255	.210	(-0.653, 2.969)	3.183	(0.521, 19.467)

Reference categories are as follows: Demonstrative: distal term, Position of addressee: side-by-side; Region: Region 1;
*=p <.05; ** = p <.01; *** = p< .001

Supplementary Table 31. Classification table for the *a priori* Italian model, overall percentage correct: 95.7%

		Predicted	
		Proximal	Distal
Observed	Proximal	343	35
		90.70%	9.30%
	Distal	11	691
		1.60%	98.40%

Supplementary Table 32. Fixed coefficients for the *a priori* Italian GLMM (multilevel model).

	Coefficient	Std. Error	t-value	p-value	95% Confidence Interval	Exp(Coefficient)	95% Confidence Interval for Exp(Coefficient)
Distal term: Intercept	-4.196***	0.452	-9.274	<.001	(-5.089, -3.302)	0.015	(0.006, 0.037)
Position opposite	1.099	0.581	1.892	.059	(-0.041, 2.239)	3.002	(0.96, 9.387)
Region							
Region 3	9.196***	1.272	7.227	<.001	(6.699, 11.692)	9856.54	(811.848, 119666.902)
Region 2	7.247***	0.546	13.27	<.001	(6.175, 8.318)	1403.476	(480.659, 4098.012)
Interactions:							
Opp × Region 3	-1.825	1.02	-1.789	.074	(-3.826, 0.177)	0.161	(0.022, 1.193)
Opp × Region 2	-1.521**	0.567	-2.681	.007	(-2.635, -0.408)	0.218	(0.072, 0.665)

Reference categories are as follows: Demonstrative: proximal term, Position of addressee: side-by-side; Region: Region 1;
*=p <.05; ** = p <.01; *** = p< .001

Supplementary Table 33. Classification table for the *a priori* Japanese model, overall percentage correct: 88.6%

		Predicted		
		Proximal	Distal	3rd term
Observed	Proximal	383	10	3
		96.70%	2.50%	0.80%
	Distal	6	397	46
		1.30%	88.40%	10.20%
	3rd term	19	55	305
		5.00%	14.50%	80.50%

Supplementary Table 34. Fixed coefficients for the *a priori* Japanese GLMM (multilevel model).

	Coefficient	Std. Error	t-value	p-value	95% Confidence Interval	Exp(Coefficient)	95% Confidence Interval for Exp(Coefficient)
Distal term:							
Intercept	-5.183***	1.063	-4.874	<.001	(-7.269, -3.097)	0.006	(0.001, 0.045)
Position opposite	0.006	0.51	0.013	.990	(-0.993, 1.006)	1.006	(0.37, 2.735)
Region							
Region 3	9.818***	1.62	6.062	<.001	(6.64, 12.996)	18364.277	(765.459, 440581.046)
Region 2	7.897***	1.849	4.272	<.001	(4.27, 11.524)	2689.08	(71.531, 101091.673)
Interactions:							
Opp × Region 3	-0.334	1.105	-0.302	.762	(-2.503, 1.834)	0.716	(0.082, 6.26)
Opp × Region 2	-0.361	0.875	-0.413	.680	(-2.077, 1.354)	0.697	(0.125, 3.874)
3rd term:							
Intercept	-3.143***	0.478	-6.57	<.001	(-4.082, -2.205)	0.043	(0.017, 0.11)
Position opposite	0.112	0.259	0.431	.667	(-0.397, 0.62)	1.118	(0.672, 1.859)
Region							
Region 3	4.955***	0.948	5.226	<.001	(3.095, 6.815)	141.838	(22.08, 911.15)
Region 2	6.569***	1.115	5.891	<.001	(4.381, 8.756)	712.324	(79.904, 6350.182)
Interactions:							
Opp × Region 3	1.603	0.843	1.903	.057	(-0.05, 3.257)	4.97	(0.951, 25.965)
Opp × Region 2	0.018	0.773	0.023	.981	(-1.498, 1.535)	1.018	(0.223, 4.639)

Reference categories are as follows: Demonstrative: proximal term, Position of addressee: side-by-side; Region: Region 1;
*=p <.05; ** = p <.01; *** = p< .001

Supplementary Table 35. Classification table for the follow-up Korean model, overall percentage correct: 87.0%

		Predicted		
		Proximal	Distal	3rd term
Observed	Proximal	0	25	8
		0.00%	75.80%	24.20%
	Distal	0	634	27
		0.00%	95.90%	4.10%
	3rd term	0	43	55
		0.00%	43.90%	56.10%

Supplementary Table 36. Fixed coefficients for the follow-up Korean GLMM (multilevel model) (Excluding Region 1).

	Coefficient	Std. Error	t-value	p-value	95% Confidence Interval	Exp(Coefficient)	95% Confidence Interval for Exp(Coefficient)
Distal term: Intercept	3.929***	0.862	4.558	<.001	(2.237, 5.621)	50.839	(9.362, 276.077)
Position opposite	-1.14	0.959	-1.189	.235	(-3.021, 0.742)	0.32	(0.049, 2.101)
Region 3	0.062	1.003	0.062	.951	(-1.907, 2.031)	1.064	(0.149, 7.623)
Opp*Region 3	-0.06	1.023	-0.058	.954	(-2.067, 1.948)	0.942	(0.127, 7.012)
3rd term: Intercept	-0.171	1.018	-0.168	.867	(-2.171, 1.829)	0.843	(0.114, 6.227)
Position opposite	0.133	1.03	0.129	.897	(-1.889, 2.155)	1.142	(0.151, 8.626)
Region 3	-0.82	1.133	-0.724	.469	(-3.045, 1.404)	0.44	(0.048, 4.07)
Opp*Region 3	1.342	1.386	0.968	.333	(-1.379, 4.063)	3.826	(0.252, 58.133)

Reference categories are as follows: Demonstrative: proximal term, Position of addressee: side-by-side; Region: Region 2;
*=p <.05; ** = p <.01; *** = p< .001

Supplementary Table 37. Classification table for the *a priori* Latvian model, overall percentage correct: 88.9%

		Predicted	
		Proximal	Distal
Observed	Proximal	533	55
		90.60%	9.40%
	Distal	73	491
		12.90%	87.10%

Supplementary Table 38. Fixed coefficients for the *a priori* Latvian GLMM (multilevel model).

	Coefficient	Std. Error	t-value	p-value	95% Confidence Interval	Exp(Coefficient)	95% Confidence Interval for Exp(Coefficient)
Distal term: Intercept	-2.21**	0.622	-3.553	<.001	(-3.467, -0.954)	0.11	(0.031, 0.385)
Position opposite	-0.143	0.356	-0.403	.687	(-0.841, 0.555)	0.867	(0.431, 1.741)
Region							
Region 3	3.743***	0.688	5.445	<.001	(2.394, 5.092)	42.217	(10.958, 162.656)
Region 2	2.587***	0.574	4.51	<.001	(1.462, 3.713)	13.292	(4.313, 40.967)
Interactions:							
Opp × Region 3	-0.287	0.413	-0.693	.488	(-1.098, 0.524)	0.751	(0.334, 1.69)
Opp × Region 2	-0.297	0.368	-0.808	.419	(-1.018, 0.424)	0.743	(0.361, 1.528)

Reference categories are as follows: Demonstrative: proximal term, Position of addressee: side-by-side; Region: Region 1;
*=p <.05; ** = p <.01; *** = p< .001

Supplementary Table 39. Classification table for the *a priori* Lithuanian model, overall percentage correct: 85.1%

		Predicted		
		Proximal	Distal	3rd term
Observed	Proximal	433	59	5
		87.10%	11.90%	1.00%
	Distal	34	477	21
		6.40%	89.70%	3.90%
	3rd term	13	40	70
		10.60%	32.50%	56.90%

Supplementary Table 40. Fixed coefficients for the *a priori* Lithuanian GLMM (multilevel model).

	Coefficient	Std. Error	t-value	p-value	95% Confidence Interval	Exp(Coefficient)	95% Confidence Interval for Exp(Coefficient)
Distal term:							
Intercept	-2.672***	0.539	-4.954	<.001	(-3.754, -1.589)	0.069	(0.023, 0.204)
Position opposite	-1.618**	0.549	-2.95	.003	(-2.694, -0.542)	0.198	(0.068, 0.582)
Region							
Region 3	4.866***	0.844	5.764	<.001	(3.21, 6.522)	129.807	(24.773, 680.174)
Region 2	3.715***	0.592	6.273	<.001	(2.553, 4.877)	41.065	(12.847, 131.265)
Interactions:							
Opp × Region 3	2.357**	0.765	3.08	.002	(0.855, 3.858)	10.554	(2.352, 47.367)
Opp × Region 2	1.662*	0.678	2.45	.014	(0.331, 2.993)	5.27	(1.393, 19.945)
3rd term:							
Intercept	-5.168***	1.072	-4.821	<.001	(-7.274, -3.063)	0.006	(0.001, 0.047)
Position opposite	-0.103	0.412	-0.249	.803	(-0.911, 0.706)	0.902	(0.402, 2.026)
Region							
Region 3	5.887***	1.411	4.171	<.001	(3.118, 8.656)	360.341	(22.597, 5746.141)
Region 2	2.393*	1.13	2.119	.034	(0.177, 4.61)	10.949	(1.193, 100.457)
Interactions:							
Opp × Region 3	0.215	0.588	0.365	.715	(-0.94, 1.369)	1.239	(0.391, 3.932)
Opp × Region 2	-0.249	0.712	-0.35	.726	(-1.646, 1.147)	0.779	(0.193, 3.149)

Reference categories are as follows: Demonstrative: proximal term, Position of addressee: side-by-side; Region: Region 1;
*=p <.05; ** = p <.01; *** = p< .001

Supplementary Table *41*. Classification table for the *a priori* Maltese model, overall percentage correct: 94.4%

		Predicted	
		Proximal	Distal
Observed	Proximal	357	36
		90.80%	9.20%
	Distal	33	798
		4.00%	96.00%

Supplementary Table *42*. Fixed coefficients for the *a priori* Maltese GLMM (multilevel model).

	Coefficient	Std. Error	t-value	p-value	95% Confidence Interval	Exp(Coefficient)	95% Confidence Interval for Exp(Coefficient)
Distal term: Intercept	-2.001*	0.876	-2.283	.030	(-3.794, -0.207)	0.135	(0.022, 0.813)
Position opposite	-0.665	0.559	-1.189	.235	(-1.763, 0.432)	0.514	(0.172, 1.541)
Region							
Region 3	8.748***	0.902	9.701	<.001	(6.979, 10.517)	6295.94	(1073.42, 36927.651)
Region 2	6.4***	0.709	9.028	<.001	(5.009, 7.79)	601.582	(149.735, 2416.94)
Interactions:							
Opp × Region 3	-0.507	1.3	-0.39	.697	(-3.056, 2.043)	0.602	(0.047, 7.71)
Opp × Region 2	0.01	0.891	0.011	.991	(-1.739, 1.758)	1.01	(0.176, 5.802)

Reference categories are as follows: Demonstrative: proximal term, Position of addressee: side-by-side; Region: Region 1;
*=p <.05; ** = p <.01; *** = p< .001

Supplementary Table 43. Classification table for the *a priori* Mandarin model, overall percentage correct: 91.3%

		Predicted	
		Proximal	Distal
Observed	Proximal	400	75
		84.20%	15.80%
	Distal	32	717
		4.30%	95.70%

Supplementary Table 44. Fixed coefficients for the *a priori* Mandarin GLMM (multilevel model).

	Coefficient	Std. Error	t-value	p-value	95% Confidence Interval	Exp(Coefficient)	95% Confidence Interval for Exp(Coefficient)
Distal term: Intercept	-3.347***	0.318	-10.529	<.001	(-3.974, -2.719)	0.035	(0.019, 0.066)
Position opposite	0.878	0.472	1.858	.063	(-0.049, 1.804)	2.405	(0.952, 6.077)
Region							
Region 3	7.138***	0.672	10.627	<.001	(5.821, 8.456)	1259.38	(337.144, 4704.334)
Region 2	5.328***	0.432	12.33	<.001	(4.48, 6.176)	205.986	(88.243, 480.838)
Interactions:							
Opp × Region 3	-1.026	0.931	-1.102	.271	(-2.853, 0.801)	0.358	(0.058, 2.228)
Opp × Region 2	-1.579**	0.554	-2.848	.004	(-2.666, -0.491)	0.206	(0.07, 0.612)

Reference categories are as follows: Demonstrative: proximal term, Position of addressee: side-by-side; Region: Region 1;
*=p <.05; ** = p <.01; *** = p< .001

Supplementary Table 45. Classification table for the *a priori* Marathi model, overall percentage correct: 86.5%

		Predicted	
		Proximal	Distal
Observed	Proximal	323	66
		83.00%	17.00%
	Distal	31	300
		9.40%	90.60%

Supplementary Table 46. Fixed coefficients for the *a priori* Marathi GLMM (multilevel model).

	Coefficient	Std. Error	t-value	p-value	95% Confidence Interval	Exp(Coefficient)	95% Confidence Interval for Exp(Coefficient)
Distal term: Intercept	-2.673***	0.704	-3.795	<.001	(-4.1, -1.246)	0.069	(0.017, 0.288)
Position opposite	0.178	0.353	0.505	.614	(-0.515, 0.872)	1.195	(0.597, 2.392)
Region							
Region 3	3.818***	0.743	5.136	<.001	(2.358, 5.277)	45.502	(10.573, 195.819)
Region 2	2.886***	0.584	4.94	<.001	(1.739, 4.033)	17.924	(5.692, 56.441)
Interactions:							
Opp × Region 3	-0.475	0.521	-0.912	.362	(-1.497, 0.548)	0.622	(0.224, 1.729)
Opp × Region 2	-0.568	0.408	-1.392	.164	(-1.37, 0.233)	0.566	(0.254, 1.263)

Reference categories are as follows: Demonstrative: proximal term, Position of addressee: side-by-side; Region: Region 1;
*=p <.05; ** = p <.01; *** = p< .001

Supplementary Table 47. Classification table for the *a priori* Nepali model, overall percentage correct: 92.1%

		Predicted	
		Proximal	Distal
Observed	Proximal	370	77
		82.80%	17.20%
	Distal	14	691
		2.00%	98.00%

Supplementary Table 48. Fixed coefficients for the *a priori* Nepali GLMM (multilevel model).

	Coefficient	Std. Error	t-value	p-value	95% Confidence Interval	Exp(Coefficient)	95% Confidence Interval for Exp(Coefficient)
Distal term: Intercept	-4.519***	0.679	-6.66	<.001	(-5.853, -3.186)	0.011	(0.003, 0.041)
Position opposite	0.698	0.655	1.065	.287	(-0.588, 1.983)	2.009	(0.556, 7.264)
Region							
Region 3	9.754***	0.932	10.471	<.001	(7.926, 11.582)	17221.947	(2769.118, 107108.282)
Region 2	6.414***	0.621	10.33	<.001	(5.196, 7.633)	610.542	(180.548, 2064.607)
Interactions:							
Opp × Region 3	0.006	1.405	0.005	.996	(-2.749, 2.762)	1.007	(0.064, 15.836)
Opp × Region 2	-0.698	0.679	-1.027	.304	(-2.03, 0.634)	0.498	(0.131, 1.886)

Reference categories are as follows: Demonstrative: proximal term, Position of addressee: side-by-side; Region: Region 1;
*=p <.05; ** = p <.01; *** = p< .001

Supplementary Table 49. Classification table for the *a priori* Norwegian model,
overall percentage correct: 95.3%

		Predicted	
		Proximal	Distal
Observed	Proximal	110	29
		79.10%	20.90%
	Distal	10	679
		1.50%	98.50%

Supplementary Table 50. Fixed coefficients for the *a priori* Norwegian GLMM (multilevel model).

	Coefficient	Std. Error	t-value	p-value	95% Confidence Interval	Exp(Coefficient)	95% Confidence Interval for Exp(Coefficient)
Distal term: Intercept	1.344	0.835	1.608	.123	(-0.395, 3.082)	3.833	(0.674, 21.798)
Position opposite	-0.164	0.47	-0.349	.727	(-1.086, 0.758)	0.849	(0.338, 2.133)
Region							
Region 3	4.236***	0.584	7.254	<.001	(3.09, 5.382)	69.142	(21.976, 217.54)
Region 2	3.389***	0.706	4.803	<.001	(2.004, 4.774)	29.634	(7.419, 118.367)
Interactions:							
Opp × Region 3	-0.309	0.369	-0.837	.403	(-1.032, 0.415)	0.734	(0.356, 1.514)
Opp × Region 2	0.164	1.1	0.149	.882	(-1.996, 2.324)	1.178	(0.136, 10.217)

Reference categories are as follows: Demonstrative: proximal term, Position of addressee: side-by-side; Region: Region 1;
*=p <.05; ** = p <.01; *** = p< .001

Supplementary Table 51. Classification table for the *a priori* Tseltal model, overall percentage correct: 86.1%

		Predicted	
		Proximal	Distal
Observed	Proximal	221	53
		80.70%	19.30%
	Distal	37	336
		9.90%	90.10%

Supplementary Table 52. Fixed coefficients for the *a priori* Tseltal GLMM (multilevel model).

	Coefficient	Std. Error	t-value	p-value	95% Confidence Interval	Exp(Coefficient)	95% Confidence Interval for Exp(Coefficient)
Distal term: Intercept	-2.225***	0.375	-5.938	<.001	(-2.974, -1.476)	0.108	(0.051, 0.228)
Position opposite	0.171	0.53	0.322	.748	(-0.871, 1.212)	1.186	(0.419, 3.359)
Region							
Region 3	3.933***	0.634	6.205	<.001	(2.688, 5.177)	51.048	(14.705, 177.211)
Region 2	3.661***	0.64	5.719	<.001	(2.404, 4.917)	38.884	(11.065, 136.648)
Interactions:							
Opp × Region 3	0.662	0.685	0.966	.334	(-0.683, 2.006)	1.938	(0.505, 7.434)
Opp × Region 2	-0.588	0.642	-0.917	.360	(-1.849, 0.672)	0.555	(0.157, 1.958)

Reference categories are as follows: Demonstrative: proximal term, Position of addressee: side-by-side; Region: Region 1;
*=p <.05; ** = p <.01; *** = p< .001

Supplementary Table 53. Classification table for the *a priori* Turkish model, overall percentage correct: 85.3%

		Predicted		
		Proximal	Distal	3rd term
Observed	Proximal	478	64	3
		87.70%	11.70%	0.60%
	Distal	24	546	7
		4.20%	94.60%	1.20%
	3rd term	38	44	20
		37.30%	43.10%	19.60%

Supplementary Table 54. Fixed coefficients for the a priori Turkish GLMM (multilevel model).

	Coefficient	Std. Error	t-value	p-value	95% Confidence Interval	Exp(Coefficient)	95% Confidence Interval for Exp(Coefficient)
Distal term:							
Intercept	-4.421***	0.652	-6.781	<.001	(-5.702, -3.14)	0.012	(0.003, 0.043)
Position opposite	-1.634	0.996	-1.641	.101	(-3.588, 0.32)	0.195	(0.028, 1.377)
Region							
Region 3	7.205***	0.842	8.562	<.001	(5.554, 8.856)	1346.425	(258.332, 7017.561)
Region 2	5.502***	0.775	7.104	<.001	(3.983, 7.021)	245.179	(53.655, 1120.366)
Interactions:							
Opp × Region 3	1.635	1.133	1.442	.149	(-0.589, 3.858)	5.129	(0.555, 47.389)
Opp × Region 2	1.029	1.039	0.99	.322	(-1.01, 3.068)	2.798	(0.364, 21.499)
3rd term:							
Intercept	-5.298***	0.766	-6.914	<.001	(-6.801, -3.794)	0.005	(0.001, 0.023)
Position opposite	1.144	0.736	1.555	.120	(-0.299, 2.588)	3.141	(0.742, 13.301)
Region							
Region 3	5.027***	0.872	5.765	<.001	(3.317, 6.738)	152.545	(27.565, 844.196)
Region 2	4.219***	0.855	4.934	<.001	(2.542, 5.897)	67.992	(12.702, 363.951)
Interactions:							
Opp × Region 3	-1.64*	0.78	-2.102	.036	(-3.171, -0.11)	0.194	(0.042, 0.896)
Opp × Region 2	-1.501*	0.698	-2.152	.032	(-2.871, -0.132)	0.223	(0.057, 0.876)

Reference categories are as follows: Demonstrative: proximal term, Position of addressee: side-by-side; Region: Region 1;
*=p <.05; ** = p <.01; *** = p< .001

Supplementary Table 55. Classification table for the *a priori* Vietnamese model, overall percentage correct: 84.5%

		Predicted		
		Proximal	Distal	3rd term
Observed	Proximal	386	27	7
		91.90%	6.40%	1.70%
	Distal	13	465	45
		2.50%	88.90%	8.60%
	3rd term	7	70	73
		4.70%	46.70%	48.70%

Supplementary Table 56. Fixed coefficients for the *a priori* Vietnamese GLMM (multilevel model).

	Coefficient	Std. Error	t-value	p-value	95% Confidence Interval	Exp(Coefficient)	95% Confidence Interval for Exp(Coefficient)
Distal term:							
Intercept	-3.768***	0.6	-6.283	<.001	(-4.964, -2.573)	0.023	(0.007, 0.076)
Position opposite	-0.753	0.782	-0.964	.335	(-2.286, 0.78)	0.471	(0.102, 2.182)
Region							
Region 3	7.82***	0.779	10.036	<.001	(6.291, 9.349)	2490.917	(539.934, 11491.527)
Region 2	6.048***	0.773	7.82	<.001	(4.53, 7.565)	423.235	(92.8, 1930.262)
Interactions:							
Opp × Region 3	-0.131	0.77	-0.17	.865	(-1.642, 1.381)	0.877	(0.194, 3.978)
Opp × Region 2	0.103	0.833	0.124	.901	(-1.53, 1.737)	1.109	(0.217, 5.68)
3rd term:							
Intercept	-5.813***	0.857	-6.786	<.001	(-7.502, -4.125)	0.003	(0.001, 0.016)
Position opposite	0.959	0.702	1.367	.172	(-0.417, 2.336)	2.61	(0.659, 10.341)
Region							
Region 3	7.109***	0.994	7.15	<.001	(5.158, 9.059)	1222.353	(173.787, 8597.602)
Region 2	6.101***	0.866	7.045	<.001	(4.401, 7.8)	446.112	(81.57, 2439.823)
Interactions:							
Opp × Region 3	-1.391	0.949	-1.466	.143	(-3.253, 0.47)	0.249	(0.039, 1.601)
Opp × Region 2	-1.618*	0.649	-2.493	.013	(-2.892, -0.345)	0.198	(0.055, 0.708)

Reference categories are as follows: Demonstrative: proximal term, Position of addressee: side-by-side; Region: Region 1;
*=p <.05; ** = p <.01; *** = p< .001

Supplementary Table 57. Classification table for the *a priori* Võro model, overall percentage correct: 87.0%

		Predicted		
		Proximal	Distal	3rd term
Observed	Proximal	230	23	13
		86.50%	8.60%	4.90%
	Distal	16	545	12
		2.80%	95.10%	2.10%
	3rd term	30	32	71
		22.60%	24.10%	53.40%

Supplementary Table 58. Fixed coefficients for the *a priori* Võro GLMM (multilevel model).

	Coefficient	Std. Error	t-value	p-value	95% Confidence Interval	Exp(Coefficient)	95% Confidence Interval for Exp(Coefficient)
Distal term:							
Intercept	-2.889***	0.584	-4.949	<.001	(-4.046, -1.733)	0.056	(0.017, 0.177)
Position opposite	-1.063	0.806	-1.319	.188	(-2.646, 0.519)	0.345	(0.071, 1.68)
Region							
Region 3	8.396***	1.001	8.386	<.001	(6.431, 10.36)	4427.445	(620.699, 31580.974)
Region 2	6.5***	0.83	7.829	<.001	(4.87, 8.129)	664.94	(130.376, 3391.322)
Interactions:							
Opp × Region 3	1.048	0.969	1.082	.280	(-0.853, 2.95)	2.853	(0.426, 19.101)
Opp × Region 2	0.226	1.045	0.217	.828	(-1.824, 2.277)	1.254	(0.161, 9.749)
3rd term:							
Intercept	-1.438**	0.482	-2.983	.004	(-2.407, -0.469)	0.237	(0.09, 0.626)
Position opposite	-0.714	0.439	-1.627	.104	(-1.575, 0.147)	0.49	(0.207, 1.158)
Region							
Region 3	2.572*	0.997	2.58	.010	(0.616, 4.529)	13.098	(1.851, 92.663)
Region 2	2.16**	0.805	2.682	.007	(0.579, 3.74)	8.67	(1.785, 42.11)
Interactions:							
Opp × Region 3	0.855	0.987	0.867	.386	(-1.082, 2.792)	2.352	(0.339, 16.321)
Opp × Region 2	0.541	0.662	0.818	.414	(-0.757, 1.839)	1.718	(0.469, 6.292)

Reference categories are as follows: Demonstrative: proximal term, Position of addressee: side-by-side; Region: Region 1;
*=p <.05; ** = p <.01; *** = p< .001

Supplementary Table 59. Classification table for the follow-up Yucatec model, overall percentage correct: 92.2%

		Predicted	
		Proximal	Distal
Observed	Proximal	64	46
		58.20%	41.8%
	Distal	13	378
		3.3%	96.70%

Supplementary Table 60. Fixed coefficients for the follow-up Yucatec GLMM (multilevel model) (excluding Region 3).

	Coefficient	Std. Error	t-value	p-value	95% Confidence Interval	Exp(Coefficient)	95% Confidence Interval for Exp(Coefficient)
Distal term: Intercept	1.152	0.61	1.888	.073	(-0.117, 2.42)	3.164	(0.89, 11.248)
Position opposite	0.232	0.332	0.7	.484	(-0.42, 0.884)	1.261	(0.657, 2.42)
Region 2	2.441***	0.636	3.839	<.001	(1.192, 3.69)	11.48	(3.293, 40.029)
Opp*Region 2	-0.057	0.611	-0.094	.925	(-1.257, 1.143)	0.944	(0.284, 3.136)

Reference categories are as follows: Demonstrative: proximal term, Position of addressee: side-by-side; Region: Region 1;
*=p <.05; ** = p <.01; *** = p< .001

Supplementary Table 61. Classification table for the *a priori* Tunisian Arabic model, overall percentage correct: 93.1%

		Predicted		
		Proximal	Distal	3rd term
Observed	Proximal	402	43	2
		89.90%	9.60%	0.40%
	Distal	5	653	10
		0.70%	97.80%	1.50%
	3rd term	1	24	84
		0.90%	22.00%	77.10%

Supplementary Table 62. Fixed coefficients for the *a priori* Tunisian Arabic GLMM (multilevel model).

	Coefficient	Std. Error	t-value	p-value	95% Confidence Interval	Exp(Coefficient)	95% Confidence Interval for Exp(Coefficient)
Distal term:							
Intercept	-4.548***	0.571	-7.967	<.001	(-5.669, -3.426)	0.011	(0.003, 0.033)
Position opposite	-0.416	0.416	-1.002	.317	(-1.232, 0.399)	0.659	(0.292, 1.49)
Region							
Region 3	9.983***	1.47	6.791	<.001	(7.099, 12.868)	21662.73	(1210.821, 387566.513)
Region 2	6.735***	0.623	10.816	<.001	(5.513, 7.957)	841.294	(247.962, 2854.369)
Interactions:							
Opp × Region 3	-1.015	0.91	-1.115	.265	(-2.8, 0.771)	0.363	(0.061, 2.161)
Opp × Region 2	0.477	0.631	0.756	.450	(-0.761, 1.715)	1.611	(0.467, 5.559)
3rd term:							
Intercept	-8.136***	1.42	-5.731	<.001	(-10.922, -5.349)	0	(0.00001806, 0.005)
Position opposite	-12.323***	1.143	-10.786	<.001	(-14.564, -10.081)	0.000004449	(0.0000004729, 0.00004185)
Region							
Region 3	9.882***	1.765	5.598	<.001	(6.418, 13.346)	19574.624	(613.026, 625039.86)
Region 2	6.288***	1.537	4.092	<.001	(3.273, 9.302)	537.878	(26.388, 10963.684)
Interactions:							
Opp × Region 3	11.036***	1.494	7.388	<.001	(8.105, 13.967)	62077.991	(3311.994, 1163551.905)
Opp × Region 2	13.221***	1.387	9.535	<.001	(10.501, 15.942)	552025.013	(36351.717, 8382867.277)

Reference categories are as follows: Demonstrative: proximal term, Position of addressee: side-by-side; Region: Region 1;
*=p <.05; ** = p <.01; *** = p< .001

Supplementary Table 63. Classification table for the *a priori* Basque model, overall percentage correct: 88.8%

		Predicted		
		Proximal	Distal	3rd term
Observed	Proximal	376	19	0
		95.20%	4.80%	0.00%
	Distal	13	538	61
		2.10%	87.90%	10.00%
	3rd term	1	43	173
		0.50%	19.80%	79.70%

Supplementary Table 64. Fixed coefficients for the *a priori* Basque GLMM (multilevel model).

	Coefficient	Std. Error	t-value	p-value	95% Confidence Interval	Exp(Coefficient)	95% Confidence Interval for Exp(Coefficient)
Distal term:							
Intercept	-3.705***	0.47	-7.89	<.001	(-4.641, -2.769)	0.025	(0.01, 0.063)
Position opposite	-0.386	0.425	-0.908	.364	(-1.221, 0.448)	0.68	(0.295, 1.566)
Region							
Region 3	8.787***	1.11	7.916	<.001	(6.609, 10.964)	6546.289	(741.626, 57783.67)
Region 2	7.986***	0.675	11.836	<.001	(6.662, 9.31)	2940.379	(782.482, 11049.231)
Interactions:							
Opp × Region 3	0.628	1.521	0.413	.680	(-2.356, 3.611)	1.874	(0.095, 37.019)
Opp × Region 2	-0.072	0.735	-0.098	.922	(-1.515, 1.371)	0.931	(0.22, 3.938)
3rd term:							
Intercept	-17.485***	0.235	-74.405	<.001	(-18.131, -16.839)	2.548E-08	(0.00000001336, 0.00000004861)
Position opposite	11.639***	0.94	12.38	<.001	(9.795, 13.484)	113450.075	(17936.45, 717584.545)
Region							
Region 3	22.4***	1.071	20.907	<.001	(20.298, 24.502)	5349808176	(653764707.717, 43777902321.54)
Region 2	18.215***	0.491	37.102	<.001	(17.252, 19.178)	81397402.76	(31067064.909, 213265630.178)
Interactions:							
Opp × Region 3	-12.009***	1.706	-7.038	<.001	(-15.356, -8.661)	0.000006092	(0.0000002143, 0)
Opp × Region 2	-12.786***	1.239	-10.32	<.001	(-15.217, -10.355)	0.0000028	(0.0000002463, 0.00003183)

Reference categories are as follows: Demonstrative: proximal term, Position of addressee: side-by-side; Region: Region 1;
*=p <.05; ** = p <.01; *** = p< .001

Supplementary Table 65. Classification table for the *a priori* Bulgarian model, overall percentage correct: 97.6%

		Predicted	
		Proximal	Distal
Observed	Proximal	420	6
		98.60%	1.40%
	Distal	12	318
		3.60%	96.40%

Supplementary Table 66. Fixed coefficients for the *a priori* Bulgarian GLMM (multilevel model).

	Coefficient	Std. Error	t-value	p-value	95% Confidence Interval	Exp(Coefficient)	95% Confidence Interval for Exp(Coefficient)
Distal term: Intercept	-7.543***	1.163	-6.486	<.001	(-9.93, -5.155)	0.001	(0.00004869, 0.006)
Position opposite	-11.991***	0.557	-21.516	<.001	(-13.085, -10.897)	0.000006202	(0.000002077, 0.00001852)
Region							
Region 3	12.246***	1.874	6.534	<.001	(8.567, 15.926)	208189.052	(5253.093, 8250887.934)
Region 2	7.362***	1.217	6.052	<.001	(4.974, 9.751)	1575.733	(144.625, 17168.047)
Interactions:							
Opp × Region 3	10.747***	0.749	14.357	<.001	(9.277, 12.217)	46492.768	(10694.484, 202120.773)
Opp × Region 2	11.453***	0.7	16.364	<.001	(10.079, 12.827)	94209.81	(23843.667, 372236.715)

Reference categories are as follows: Demonstrative: proximal term, Position of addressee: side-by-side; Region: Region 1;
*=p <.05; ** = p <.01; *** = p< .001

Supplementary Table 67. Classification table for the *a priori* Castilian model, overall percentage correct: 94.7%

		Predicted		
		Proximal	Distal	3rd term
Observed	Proximal	339	0	8
		97.70%	0.00%	2.30%
	Distal	0	290	24
		0.00%	92.40%	7.60%
	3rd term	9	16	394
		2.10%	3.80%	94.00%

Supplementary Table *68*. Fixed coefficients for the *a priori* Castilian GLMM (multilevel model).

	Coefficient	Std. Error	t-value	p-value	95% Confidence Interval	Exp(Coefficient)	95% Confidence Interval for Exp(Coefficient)
Distal term:							
Intercept	-20.793*	0.403	-51.658	.013	(-25.96, -15.627)	9.323E-10	(0.000000000005317, 0.0000001635)
Position opposite	0.001	.	.	.	.	1.001	(0, 0)
Region							
Region 3	39.936	0.281	142.043	.122	(-474.708, 554.579)	2.20735E+17	(6.871E-207, 7.091E+240)
Region 2	22.215***	0.762	29.162	<.001	(20.624, 23.806)	4445948665	(905720326.656, 21824021117.173)
Interactions:							
Opp × Region 3	0.123	0.025	4.874	1.000	(0.102, 0.144)	1.131	(1.108, 1.155)
Opp × Region 2	-0.485	0.712	-0.681	.596	(-1.883, 0.913)	0.616	(0.152, 2.491)
3rd term:							
Intercept	-4.242***	0.689	-6.157	<.001	(-5.605, -2.879)	0.014	(0.004, 0.056)
Position opposite	0.209	0.562	0.372	.710	(-0.894, 1.312)	1.233	(0.409, 3.714)
Region							
Region 3	21.639	0.634	34.144	1.000	(-211.357, 254.636)	2499265593	(1.617E-92, 3.863E+110)
Region 2	9.328***	0.885	10.536	<.001	(7.591, 11.065)	11247.516	(1979.818, 63898.093)
Interactions:							
Opp × Region 3	-0.601	0.626	-0.96	1.000	(-774.406, 773.204)	0.548	(0, .)
Opp × Region 2	-0.802	0.881	-0.91	1.000	(-285.947, 284.343)	0.448	(6.526E-125, 3.079E+123)

Reference categories are as follows: Demonstrative: proximal term, Position of addressee: side-by-side; Region: Region 1;
*=p <.05; ** = p <.01; *** = p< .001

Supplementary Table 69. Classification table for the *a priori* Catalan model, overall percentage correct: 91.4%

		Predicted		
		Proximal	Distal	3rd term
Observed	Proximal	595	30	21
		92.1%	4.6%	3.3%
	Distal	12	513	22
		2.2%	93.8%	4.0%
	3rd term	11	15	77
		10.70%	14.6%	74.8%

Supplementary Table 70. Fixed coefficients for the *a priori* Catalan GLMM (multilevel model).

	Coefficient	Std. Error	t-value	p-value	95% Confidence Interval	Exp(Coefficient)	95% Confidence Interval for Exp(Coefficient)
Distal term:							
Intercept	-19.976***	0.493	-40.519	<.001	(-21.059, -18.894)	2.111E-09	(0.000000000715, 0.000000006232)
Position opposite	0.002	0.073	0.033	.974	(-0.14, 0.145)	1.002	(0.869, 1.156)
Region							
Region 2	23.743***	0.618	38.421	<.001	(22.53, 24.955)	20478836423	(6089412271.542, 68870807648.418)
Region 3	20.056***	0.23	87.342	<.001	(19.587, 20.525)	513228277.6	(321116017.909, 820274449.919)
Interactions:							
SBS × Region 2	-0.002	0.454	-0.005	.996	(-0.892, 0.887)	0.998	(0.41, 2.428)
SBS × Region 3	-0.521	0.27	-1.928	.054	(-1.051, 0.009)	0.594	(0.35, 1.009)
3rd term:							
Intercept	-5.709***	1.349	-4.232	<.001	(-8.356, -3.062)	0.003	(0, 0.047)
Position opposite	0.2	0.017	12.01	1.000	(-95.655, 96.056)	1.222	(2.868E-42, 5.206E+41)
Region							
Region 2	4.107	1.961	2.095	1.000	(-16753.835, 16762.048)	60.753	(0, .)
Region 3	3.999	1.664	2.403	1.000	(-8416.55, 8424.548)	54.535	(0, .)
Interactions:							
SBS × Region 2	-0.2	0.405	-0.495	.620	(-0.995, 0.594)	0.818	(0.37, 1.811)
SBS × Region 3	-0.709	0.419	-1.692	.091	(-1.531, 0.113)	0.492	(0.216, 1.12)

Reference categories are as follows: Demonstrative: proximal term, Position of addressee: side-by-side; Region: Region 1;
*=p <.05; ** = p <.01; *** = p< .001

Supplementary Table 71. Classification table for the *a priori* Georgian model, overall percentage correct: 90.7%

		Predicted		
		Proximal	Distal	3rd term
Observed	Proximal	332	6	18
		93.30%	1.70%	5.10%
	Distal	10	50	1
		16.40%	82.00%	1.60%
	3rd term	18	4	173
		9.20%	2.10%	88.70%

Supplementary Table 72. Fixed coefficients for the *a priori* Georgian GLMM (multilevel model).

	Coefficient	Std. Error	t-value	p-value	95% Confidence Interval	Exp(Coefficient)	95% Confidence Interval for Exp(Coefficient)
Distal term:							
Intercept	-5.762***	1.184	-4.869	<.001	(-8.093, -3.432)	0.003	(0, 0.032)
Position opposite	-12.275***	1.143	-10.742	<.001	(-14.52, -10.031)	0.000004665	(0.0000004945, 0.000044)
Region							
Region 3	2.142	1.601	1.338	.182	(-1.003, 5.287)	8.517	(0.367, 197.785)
Region 2	2.294*	0.895	2.563	.011	(0.536, 4.052)	9.914	(1.709, 57.499)
Interactions:							
Opp × Region 3	16.643***	1.313	12.677	<.001	(14.065, 19.221)	16901915.02	(1282906.284, 222677786.255)
Opp × Region 2	13.046***	0.864	15.106	<.001	(11.35, 14.742)	463257.181	(84957.381, 2526057.349)
3rd term:							
Intercept	-7.366***	1.056	-6.976	<.001	(-9.516, -5.216)	0.001	(0.00007368, 0.005)
Position opposite	-11.929***	0.455	-26.218	<.001	(-12.823, -11.036)	0.000006595	(0.000002699, 0.00001612)
Region							
Region 3	9.471***	1.015	9.332	<.001	(7.474, 11.468)	12978.621	(1761.486, 95626.439)
Region 2	7.876***	0.941	8.366	<.001	(6.022, 9.73)	2633.737	(412.473, 16817.049)
Interactions:							
Opp × Region 3	9.978*	1.036	9.631	.023	(4.031, 15.924)	21536.688	(56.295, 8239277.163)
Opp × Region 2	9.72**	0.77	12.619	.002	(7.019, 12.42)	16645.502	(1118.029, 247822.386)

Reference categories are as follows: Demonstrative: proximal term, Position of addressee: side-by-side; Region: Region 1;
*=p <.05; ** = p <.01; *** = p< .001

Supplementary Table 73. Classification table for the *a priori* German model, overall percentage correct: 83.5%

		Predicted		
		Proximal	Distal	3rd term
Observed	Proximal	360	23	9
		91.80%	5.90%	2.30%
	Distal	12	397	62
		2.50%	84.30%	13.20%
	3rd term	12	72	205
		4.20%	24.90%	70.90%

Supplementary Table 74. Fixed coefficients for the *a priori* German GLMM (multilevel model).

	Coefficient	Std. Error	t-value	p-value	95% Confidence Interval	Exp(Coefficient)	95% Confidence Interval for Exp(Coefficient)
Distal term:							
Intercept	-3.342***	0.496	-6.735	<.001	(-4.316, -2.368)	0.035	(0.013, 0.094)
Position opposite	-0.341	0.794	-0.429	<.001	(-1.898, 1.216)	0.711	(0.15, 3.375)
Region							
Region 3	19.293***	0.466	41.412	.182	(18.379, 20.207)	239301085.9	(95931265.171, 596937918.292)
Region 2	5.09***	0.524	9.719	.011	(4.063, 6.118)	162.422	(58.124, 453.877)
Interactions:							
Opp × Region 3	-11.604***	1	-11.606	<.001	(-13.566, -9.643)	0.000009127	(0.000001284, 0.00006491)
Opp × Region 2	-0.011	0.98	-0.011	<.001	(-1.934, 1.912)	0.989	(0.145, 6.768)
3rd term:							
Intercept	-3.921***	0.532	-7.365	<.001	(-4.966, -2.875)	0.02	(0.007, 0.056)
Position opposite	0.342	0.694	0.493	<.001	(-1.02, 1.704)	1.408	(0.361, 5.495)
Region							
Region 3	17.782***	0.571	31.142	<.001	(16.662, 18.902)	52803268.1	(17223015.909, 161887159.412)
Region 2	6.121***	0.64	9.558	<.001	(4.864, 7.378)	455.354	(129.606, 1599.827)
Interactions:							
Opp × Region 3	-12.099***	0.845	-14.323	.023	(-13.757, -10.442)	0.000005563	(0.00000106, 0.00002918)
Opp × Region 2	-0.765	0.881	-0.869	.002	(-2.493, 0.962)	0.465	(0.083, 2.618)

Reference categories are as follows: Demonstrative: proximal term, Position of addressee: side-by-side; Region: Region 1;
*=p <.05; ** = p <.01; *** = p< .001

Supplementary Table 75. Classification table for the *a priori* Korean model, overall percentage correct: 90.0%

		Predicted		
		Proximal	Distal	3rd term
Observed	Proximal	370	25	9
		91.60%	6.20%	2.20%
	Distal	10	642	30
		1.50%	94.10%	4.40%
	3rd term	4	41	57
		3.90%	40.20%	55.90%

Supplementary Table 76. Fixed coefficients for the *a priori* Korean GLMM (multilevel model).

	Coefficient	Std. Error	t-value	p-value	95% Confidence Interval	Exp(Coefficient)	95% Confidence Interval for Exp(Coefficient)
Distal term:							
Intercept	-3.376***	0.507	-6.662	<.001	(-4.374, -2.377)	0.034	(0.013, 0.093)
Position opposite	-0.764	0.737	-1.036	.301	(-2.211, 0.683)	0.466	(0.11, 1.98)
Region							
Region 3	7.497***	0.734	10.214	<.001	(6.057, 8.937)	1802.744	(427.083, 7609.5)
Region 2	7.422***	0.928	7.999	<.001	(5.602, 9.243)	1672.911	(270.906, 10330.645)
Interactions:							
Opp × Region 3	-0.454	1.175	-0.387	.699	(-2.759, 1.851)	0.635	(0.063, 6.364)
Opp × Region 2	-0.386	1.251	-0.309	.758	(-2.84, 2.068)	0.68	(0.058, 7.909)
3rd term:							
Intercept	-17.128***	0.41	-41.75	<.001	(-18.005, -16.25)	3.644E-08	(0.00000001515, 0.00000008765)
Position opposite	11.979***	0.714	16.771	<.001	(10.577, 13.38)	159303.043	(39232.843, 646842.224)
Region							
Region 3	16.215***	0.696	23.314	<.001	(14.85, 17.579)	11012876.37	(2813722.426, 43104268.173)
Region 2	16.994***	0.845	20.108	<.001	(15.336, 18.652)	24010378.41	(4573645.727, 126047863.287)
Interactions:							
Opp × Region 3	-10.571***	1.153	-9.17	<.001	(-12.833, -8.31)	0.00002564	(0.00000267, 0)
Opp × Region 2	-11.873***	1.29	-9.201	<.001	(-14.404, -9.341)	0.000006978	(0.0000005549, 0.00008774)

Reference categories are as follows: Demonstrative: proximal term, Position of addressee: side-by-side; Region: Region 1;
*=p <.05; ** = p <.01; *** = p< .001

Supplementary Table 77. Classification table for the *a priori* Telugu model, overall percentage correct: 91.6%

		Predicted	
		Proximal	Distal
Observed	Proximal	465	70
		86.90%	13.10%
	Distal	27	590
		4.40%	95.60%

Supplementary Table 78. Fixed coefficients for the *a priori* Telugu GLMM (multilevel model).

	Coefficient	Std. Error	t-value	p-value	95% Confidence Interval	Exp(Coefficient)	95% Confidence Interval for Exp(Coefficient)
Distal term: Intercept	-18.072***	0.195	-92.658	<.001	(-18.58, -17.564)	1.417E-08	(0.000000008523, 0.00000002354)
Position opposite	-0.00000003765	0.064	0	1.000	(-0.126, 0.126)	1	(0.881, 1.135)
Region							
Region 3	36.219***	0.035	1027.533	<.001	(36.146, 36.292)	5.36552E+15	(4986721576354360, 577308736087268[illegible]
Region 2	18.582***	0.137	136.033	<.001	(18.305, 18.859)	117496307.5	(89031986.789, 155060925.534)
Interactions:							
Opp × Region 3	0.00000003765	0.072	0	1.000	(-0.141, 0.141)	1	(0.869, 1.151)
Opp × Region 2	0.084	0.25	0.337	.758	(-0.693, 0.861)	1.088	(0.5, 2.366)

Reference categories are as follows: Demonstrative: proximal term, Position of addressee: side-by-side; Region: Region 1;
*=p <.05; ** = p <.01; *** = p< .001

Supplementary Table 79. Classification table for the *a priori* Yucatec model, overall percentage correct: 92.2%

		Predicted	
		Proximal	Distal
Observed	Proximal	64	46
		58.20%	41.80%
	Distal	13	630
		2.00%	98.00%

Supplementary Table 80. Fixed coefficients for the *a priori* Yucatec GLMM (multilevel model).

	Coefficient	Std. Error	t-value	p-value	95% Confidence Interval	Exp(Coefficient)	95% Confidence Interval for Exp(Coefficient)
Distal term: Intercept	1.152	0.61	1.888	.073	(-0.117, 2.42)	3.164	(0.89, 11.248)
Position opposite	0.232	0.332	0.7	.484	(-0.419, 0.883)	1.261	(0.658, 2.419)
Region							
Region 3	15.978***	0.307	52.11	<.001	(15.376, 16.58)	8691435.907	(4760714.202, 15867589.379)
Region 2	2.441***	0.636	3.839	<.001	(1.193, 3.689)	11.48	(3.296, 39.988)
Interactions:							
Opp × Region 3	-0.232	0.332	-0.7	.484	(-0.883, 0.419)	0.793	(0.413, 1.521)
Opp × Region 2	-0.057	0.611	-0.094	.925	(-1.256, 1.142)	0.944	(0.285, 3.133)

Reference categories are as follows: Demonstrative: proximal term, Position of addressee: side-by-side; Region: Region 1;
*=p <.05; ** = p <.01; *** = p< .001

6. Open Access Data and Analysis script

Data and analysis files will be publicly available upon publication. All files are currently available at:

https://osf.io/ush2w/?view_only=1f38fa7ae6ce4bbab456eee80615ebe4

7. SI References

1. A. Khalfaoui, *A cognitive approach to analyzing demonstratives in Tunisian Arabic* (PhD dissertation, University of Minessota (2009).
2. J. I. Hualde, J. Ortiz de Urbina, A Grammar of Basque (Berlin, Mouton de Gruyter, 2003).
3. M. Dimitrova-Vulchanova, M., O. M. Tomić, O. M.**,** *The structure of the Bulgarian and Macedonian nominal expression: Introduction* in *Investigations in the Bulgarian and Macedonian Nominal Expression*, M. Dimitrova-Vulchanova, O. M. Tomić, Eds. (Trondheim: Tapir Academic Press, 2009).
4. S. Matthews, V. Yip, *Cantonese: A comprehensive grammar* (London: Routledge, 2011).
5. P.-M. Hottenroth, *The system of local deixis in Spanish* in *Here and There. Cross-linguistic Studies on Deixis and Demonstratives, J. Weissenborn, W. Klein, Eds.* (John Benjamins, Amsterdam, 1982), pp. 133-153.
6. J. I. Hualde, *Catalan* (London: Routledge, 1992).
7. E. Hansen, L. Heltorf, *Grammatik over det Danske Sprog* (København: Det Danske Sprog- og Litteraturselskab, 2011).
8. W. Z. Shetter, *Dutch. An Essential Grammar* (London: Routledge, 1994).
9. R. Huddleston, G. K. Pullum, *The Cambridge Grammar of the English Language* (Cambridge: Cambridge University Press, 2002).
10. M. Reile, Space and demonstratives: An experiment with Estonian exophoric demonstratives, *Journal of Estonian and Finno-Ugrian Linguistics* **6(2)**, (2015).
11. R. Laury, *Demonstratives in Interaction. The emergence of a definite article in Finnish* (Amsterdam: John Benjamins, 1997).
12. B.G. Hewitt, *Georgian. A Structural Reference Grammar* (Amsterdam: John Benjamins, 1995).
13. F. da Milano, *Italian* in *Manual of Deixis in Romance Languages*, K. Jungbluth, F. da Milano Eds. (Berlin: Mouton de Gruyter, 2015), pp. 60-74.
14. I. Lee, S. R. Ramsey, *The Korean Language* (Albany. SUNY Series in Korean Studies, 2000).
15. T. Matthiassen, *A Short Grammar of Latvian* (Slavica: Ohio, 1997).
16. G. Judžentytė, Spatial deixis in Lithuanian: Demonstrative pronouns, *Valoda: Nozīme un Form* **8**, 173-193 (2017).
17. A. Borg, M. Azzopardi-Alexander, *Maltese* (London: Routledge, 1997).
18. Y. Wu, *Spatial Demonstratives in English and Chinese: Text and Cognition* (Amsterdam: John Benjamins, 2004).
19. R. V. Dhongde, K. Wali, *Marathi* (Amsterdam: John Benjamins, 2009).
20. J. Acharya, *A descriptive grammar of Nepali and an analyzed corpus* (PhD dissertation. Georgetown University, 1990).
21. U. Vindenes, *Complex demonstratives and cyclic change in Norwegian* (PhD dissertation, University of Oslo, 2017).
22. R. E. Asher, T. C. Kumari, *Malayalam* (Psychology Press, 1997).
23. P. Brown, *A sketch of the grammar of space in Tseltal* in *Grammars of Space: Exporations in Cognitive Diversity,* S. C. Levinson, D. P. Wilkins, S., Eds. (CUP, Cambridge, 2006), pp. 230-272.
24. L. T. Bui, *Vietnamese Demonstratives: A spatially-based polysemy network* (PhD dissertation, The University of Queensland, 2014).
25. R. Pajusalu, Võro demonstratives: Changing or disappearing? *Journal of Estonian and Finno-Ugrian Linguistics* **6(2)**, 167-190 (2015).
26. D. Birdsong, L. M. Gertken, M. Amengual, Bilingual Language Profile: An easy-to-use instrument to assess bilingualism, University of Texas. Web. <https://sites.la.utexas.edu/bilingual/> (2012).
27. H. Damasio, T. J. Grabowski, D. Tranel, L. I. Ponto, R. D. Hichwa, A. R. Damasio, Nearal correlates of naming actions and of naming spatial relations. *NeuroImage* **13**, 1053-1064 (2001).
28. M. I. Noordzij, S. F. Neggers, N. F. Ramsey, A. Postma, Neural correlates of locative prepositons. *Neuropsychologia* **46(5)**, 1576-1580 (2008).

29. Rocca, R., Coventry, K.R., and Tylén, K., Staib, M., Lund, T.E. & Wallentin, M. Language beyond the language system: Dorsal visuospatial pathways support processing of 1011 demonstratives and spatial language during naturalistic fMRI. *NeuroImage* **216***, 116128* (2020).
30. G. di Pellegrino, E. Làdavas, Peripersonal space in the brain. *Neuropsychologia* **66**, 126-133 (2015).
31. Y. Coello, A. Cartaud, The interrelation between peripersonal action space and interpersonal social space: Psychophysiological evidence and clinical implications. *Frontiers in Human Neuroscience* https://www.frontiersin.org/articles/10.3389/fnhum.2021.636124/full (2021).
32. A. Iriki, M. Tanaka, Y. Iwamura, Coding of modified body schema during tool use by macaque postcentral neurons. *Neuroreport* **7**, 2325-2330 (1996).
33. G. Rizzolatti, M. Matelli, G. Pavesi, Deficits in attention and movement following the removal of postarcuate (area 6) and prearcuate (area 8) cortex in macaque monkeys. *Brain* **106**, 655-673 (1983).
34. J., P. Gallivan, C. Cavina-Pratesi, J. C. Culham, Is that within reach? fMRI reveals that the human superior parieto-occipital cortex encodes objects reachable by hand. *Journal of Neuroscience* **29**, 4381-4391 (2009).
35. D. J. Quinlan, J. C. Culham, fMRI reveals a preference for near viewing in the human parietooccipital cortex. *NeuroImage* **36**, 167-187 (2007).
36. A. Cowey, M. Small, S. Ellis, Visuospatial neglect can be worse in far than in near space. *Neuropsychologia* **32**, 1059-1066.
37. P. W. Halligan, J. C. Marshall, Left neglect for near but not far space in man. *Nature* **350**, 498-500.
38. A. Berti, F. Frassinetti, When far becomes near: Remapping of space by tool use. *Journal of Cognitive Neuroscience* **12**, 415-420.
