# Supplementary material for: Spatial communication systems across languages reflect universal action constraints
Source: Nat Hum Behav. 2023 Oct 30;7(12):2099–110. doi: 10.1038/s41562-023-01697-4 (PMC10730392; doi:10.1038/s41562-023-01697-4)
Supplement: Supplementary file 2 — Reporting Summary [file 41562_2023_1697_MOESM2_ESM.pdf]

nature portfolio

Corresponding author(s): Kenny R. Coventry

Last updated by author(s): Jul 1, 2023

Reporting Summary

Nature Portfolio wishes to improve the reproducibility of the work that we publish. This form provides structure for consistency and transparency in reporting. For further information on Nature Portfolio policies, see our Editorial Policies and the Editorial Policy Checklist.

Statistics

For all statistical analyses, confirm that the following items are present in the figure legend, table legend, main text, or Methods section.

n/a	Confirmed	
☐	☒	The exact sample size (*n*) for each experimental group/condition, given as a discrete number and unit of measurement
☐	☒	A statement on whether measurements were taken from distinct samples or whether the same sample was measured repeatedly
☐	☒	The statistical test(s) used AND whether they are one- or two-sided<br>*Only common tests should be described solely by name; describe more complex techniques in the Methods section.*
☒	☐	A description of all covariates tested
☐	☒	A description of any assumptions or corrections, such as tests of normality and adjustment for multiple comparisons
☐	☒	A full description of the statistical parameters including central tendency (e.g. means) or other basic estimates (e.g. regression coefficient) AND variation (e.g. standard deviation) or associated estimates of uncertainty (e.g. confidence intervals)
☐	☒	For null hypothesis testing, the test statistic (e.g. *F*, *t*, *r*) with confidence intervals, effect sizes, degrees of freedom and *P* value noted<br>*Give P values as exact values whenever suitable.*
☒	☐	For Bayesian analysis, information on the choice of priors and Markov chain Monte Carlo settings
☐	☒	For hierarchical and complex designs, identification of the appropriate level for tests and full reporting of outcomes
☐	☒	Estimates of effect sizes (e.g. Cohen's *d*, Pearson's *r*), indicating how they were calculated

Our web collection on statistics for biologists contains articles on many of the points above.

Software and code

Policy information about availability of computer code

Data collection	Data collection was by hand - no software was used.
Data analysis	Data were analysed using bi- and multinomial multilevel modelling in SPSS version 27.

For manuscripts utilizing custom algorithms or software that are central to the research but not yet described in published literature, software must be made available to editors and reviewers. We strongly encourage code deposition in a community repository (e.g. GitHub). See the Nature Portfolio guidelines for submitting code & software for further information.

Data

Policy information about availability of data

All manuscripts must include a data availability statement. This statement should provide the following information, where applicable:

- Accession codes, unique identifiers, or web links for publicly available datasets
- A description of any restrictions on data availability
- For clinical datasets or third party data, please ensure that the statement adheres to our policy

All data and analysis scripts are available online with a url provided (also stated in the main manuscript): https://osf.io/ush2w/?view_only=1f38fa7ae6ce4bbab456eee80615ebe4

Human research participants

Policy information about studies involving human research participants and Sex and Gender in Research.

Reporting on sex and gender	The number of men and women are reported in the main manuscript (self-reported by participants); Supplementary Information Table S1 shows the number of males and females tested for each language. A priori, as stated in the main manuscript, researchers at all sites set out to test an equal number of men and women (self-reported). For the analyses we did not include sex as a variable given the goal of study was to consider general patterns across languages and the extent of variation across all participants within languages. The data are available (open source) for future analyses of data by sex.
Population characteristics	Demographic information is provided in Supplementary Information Table S1 (the mean age of the sample was 26 years, SD = 7.64). As the goal of study was to consider general patterns across languages and the extent of variation across all participants within languages, we did not examine age as a potential predictor of differences in demonstrative use. The data are available (open source) for future analyses of data by age
Recruitment	Participants took part either for nominal payment, course credit, or on a voluntary basis (commensurate with cultural norms of participation for each language). The lead researcher at each language site was responsible for recruiting participants in a culturally appropriate manner. Participants were all volunteers recruited through local advertising, word of mouth, etc. commensurate with norms for recruitment at each site. Given that participants were blind to the purpose of the study and were all native speakers (L1 speakers from birth) of the languages tested, self-selection bias is unlikely to have affected the integrity and representativeness of the data.
Ethics oversight	Prior to data collection, the study received full ethical clearance from the University of East Anglia's School of Psychology Ethics Committee (approval numbers 13-14-5 and 2017-0034-000748 granted respectively on 9/3/2015 and 8/9/2017) covering data collection across languages. Local clearance was also required for Finnish data collection (from Tartu University, approval number 293/T-21, granted on 20/5/2019). All procedures were carried out in accordance with the guidelines of the BPS, APA, APS and the Declaration of Helsinki.

Note that full information on the approval of the study protocol must also be provided in the manuscript.

Field-specific reporting

Please select the one below that is the best fit for your research. If you are not sure, read the appropriate sections before making your selection.

☐ Life sciences ☒ Behavioural & social sciences ☐ Ecological, evolutionary & environmental sciences

For a reference copy of the document with all sections, see nature.com/documents/nr-reporting-summary-flat.pdf

Behavioural & social sciences study design

All studies must disclose on these points even when the disclosure is negative.

Study description	The study is a cross-linguistic experimental quantitative study using the 'memory game method' pioneered by the lead author. The experiment measures choice of demonstrative in each of 29 languages with varied demonstrative systems manipulating the distance the reference object is from the speaker (participant) and the position of the addressee.
Research sample	Languages were selected in accordance with four working criteria: i) sampling across languages with demonstrative systems varying in number of demonstrative terms, ii) sampling between and within language families, iii) sampling across geographical areas, and iv) the availability of researchers to collect data in targeted languages. The sample of 29 languages spans geographical areas, genetic origins, and differences in spatial communication systems.<br><br>Participants for each language were all L1 speakers of the language tested.
Sampling strategy	A statistical power analysis (a priori) was performed for sample size estimation using G*Power. With power = 0.9 and an alpha = .05, and the effect sizes reported in Coventry et al.32, the projected sample size is approximately N = 17 for each language. Given the effect size was based on English, and that many languages tested have no empirical data on demonstrative production, 17 participants was set as a minimum sample size, while aiming for 30+ per language (N = 914, M = 32 participants per language).<br><br>Convenience sampling was used, while ensuring an equal balance (where possible) of male and female participants for each language (self reported). The age range of participants was broadly equivalent across languages (see Supplementary Information Table S1). Given large cultural differences across language samples, researchers were sensitive to local norms regarding the conduct of experimental work.

Data collection	The experiment employed the 'memory game' method (see references 34-36, 52, main manuscript). This established method was designed to elicit language under strictly controlled experimental conditions, but without participants being aware that language data were being collected. To do so, participants were instructed that they were taking part in a study on the effects of language on object-location memory, with memory probe trials maintaining the cover of the memory experiment throughout (see 52 for more detail).<br><br>Participants were seated at a large table (325cm long), with 12 marked locations (colored dots), spaced equidistantly 25cm apart down the midline of the table directly in front of participants, starting at 25cm from the participants' edge of the table (Supplementary Information Figure S1). On each trial, an object was placed by the experimenter (the 'addressee') on one of the colored dots. The objects placed were colored shapes on disks. All shapes were basic geometric shapes: a black cross/green star/yellow triangle/red circle/blue heart/orange square/red sun/white moon/red moon/black bar. In each language 6 objects from the set were selected (4 for Tseltal, given available colour terms), ensuring the language had a color lexicon able to differentiate the colors, and ensuring all objects were matched for gender (in gendered languages).<br><br>The experiment manipulated the distance of the object from the participant (the 'speaker' in the experiment), and the position of the addressee. The addressee was seated either next to or opposite the speaker. This addressee position manipulation was blocked and counterbalanced: the addressee switched their position once, halfway through the experiment. The distance condition was pseudo-randomized, to ensure no object or distance was used in two successive trials, preventing carry-over effects. 6 of the marked locations on the table were used, creating 3 conceptual regions: Region 1 - within the speaker's reach/peripersonal space (PPS), at 25cm and 50cm; Region 2 - out of reach for both speaker and addressee (regardless of addressee position) and at medium distance from the speaker, at 150cm and 175cm; Region 3 - at 275cm and 300cm furthest from the speaker, but in the PPS of the addressee when the addressee was seated opposite the participant.<br><br>(This information is in the main manuscript.)<br><br>Demonstrative choices were recorded using pencil and paper (ticking the demonstrative used on each given trial).<br><br>Researchers collecting the data were aware of the main manipulations in the study, but the method eliminated any potential for researchers to bias the outcome of the study.
Timing	As stated in the main manuscript. data were collected between January 2016 and December 2019, with staggered tested of languages during that period (so all testing sites used the same apparatus).
Data exclusions	Of the 914 tested, data from 40 participants were excluded based on the following a priori criteria: a) participants did not have normal or corrected to normal vision, b) participants guessed that the study was about demonstrative use, c) participants reported deliberately using demonstratives in a way they wouldn't normally use them in the study. All data exclusions took place prior to the data being submitted to independent statistical analyses.
Non-participation	No participants declined - participation was on a voluntary basis.
Randomization	The experiment was repeated measures so randomization of participants to conditions does not apply. However, trial order was psuedo-randomized (see Data Collection section above)

Reporting for specific materials, systems and methods

We require information from authors about some types of materials, experimental systems and methods used in many studies. Here, indicate whether each material, system or method listed is relevant to your study. If you are not sure if a list item applies to your research, read the appropriate section before selecting a response.

Materials & experimental systems

n/a	Involved in the study
☒	☐ Antibodies
☒	☐ Eukaryotic cell lines
☒	☐ Palaeontology and archaeology
☒	☐ Animals and other organisms
☒	☐ Clinical data
☒	☐ Dual use research of concern

Methods

n/a	Involved in the study
☒	☐ ChIP-seq
☒	☐ Flow cytometry
☒	☐ MRI-based neuroimaging
